# Development of KASP markers, SNP fingerprinting and population structure analysis of *Robinia pseudoacacia* and its closely related species

**DOI:** 10.3389/fpls.2026.1761477

**Published:** 2026-02-03

**Authors:** Haoran Wang, Ruixue Wang, Huizhong Zheng, Chunlei Zhao, Dekui Zang, Yan Ma, Fengqi Zang, Qichao Wu

**Affiliations:** 1 College of Forestry, Key Laboratory of State Forestry Administration for Silviculture of the Lower Yellow River, Shandong Agricultural University, Tai’an, China; 2 Shandong Province Forestry Protection and Development Service Center, Jinan, China

**Keywords:** DNA fingerprint, genetic diversity, KASP, *Robinia pseudoacacia*, SNP

## Abstract

*Robinia pseudoacacia* is a deciduous arbor with significant ecological and economic values. However, the current method for germplasm identification using fingerprinting primarily relies on traditional SSR markers, which suffer from limited polymorphism and cumbersome workflow. These drawbacks have hindered the development of genetic breeding in *R. pseudoacacia*. The present study aims to screen high-quality single nucleotide polymorphism (SNP) loci, develop reliable kompetitive allele-specific PCR (KASP) markers for genotyping *R. pseudoacacia* and its closely related species, further construct fingerprint profiles, and conduct genetic diversity analysis. The ultimate goal is to achieve accurate identification of *R. pseudoacacia* germplasm. In the present study, based on the single nucleotide polymorphism (SNP) loci identified through resequencing data alignment, 145 high-quality loci were screened out. Among these, 65 loci were selected for KASP marker development, and 31 core KASP markers were successfully developed ultimately. Genetic diversity and population structure of 105 *R. pseudoacacia* individuals and their related species in Daqingshan Forest Farm were analyzed using these KASP markers. The results showed that the polymorphism information content (PIC), minor allele frequency (MAF), gene diversity, and heterozygosity of the 31 core KASP markers were 0.335, 0.328, 0.428, and 0.357, respectively. The results of population structure analysis, principal component analysis (PCA), and phylogenetic tree analysis indicated that the 105 accessions could be clustered into three groups. Finally, a fingerprint profile was constructed for the 105 accessions of *R. pseudoacacia* and its closely related species based on the 31 core KASP markers, and all 105 accessions could be completely distinguished using only 12 of these markers. The KASP primers developed in this study provide a foundation for subsequent germplasm identification and genetic research of *R. pseudoacacia*.

## Introduction

*Robinia pseudoacacia*, a deciduous tree belonging to the Fabaceae family, is native to North America. Owing to its strong adaptability, fast growth rate, and diverse uses, it has been widely introduced and cultivated in many countries worldwide (Batzli et al., 1992). It was introduced into China at the end of the 19th century. After introduction, its planting area expanded rapidly, and superior clones have been selected and bred in provinces such as Shandong, Hebei, and Henan. Currently, it has become an important ecological and timber tree species (Kehrer, 2020; Ma et al., 2021). With the continuous increase in cultivated and introduced *R. pseudoacacia* germplasm, similar germplasm resources have also increased accordingly. Phenomena such as synonymy (same name for different taxa) and homonymy (different names for the same taxon) often occur, which have hindered the breeding and popularization of *R. pseudoacacia*. Currently, the identification of *R. pseudoacacia* germplasm is mainly carried out by observing morphological characteristics. However, this method has several limitations, including large planting scales, numerous observed traits, long cycles, and annual variations in traits affected by environmental factors. Additionally, the identification results are subjective (Olejnik et al., 2021). Furthermore, due to the concentrated utilization of germplasm resources and directed artificial selection, the genetic background of genetic breeding materials and cultivars of *R. pseudoacacia* tends to be narrow. Morphological identification is difficult to distinguish subtle genetic differences. Therefore, the rapid and accurate identification of *R. pseudoacacia* germplasm resources is of great significance for improving breeding efficiency, verifying cultivar authenticity, and resolving property rights disputes.

With the development of molecular biology and genomics, DNA molecular markers have been widely used in the research on genetic diversity, genetic relationship, and fingerprinting of various crops. Among them, simple sequence repeat (SSR) and single nucleotide polymorphism (SNP) markers have been prioritized for recommendation by the International Union for the Protection of New Varieties of Plants (UPOV) (Button, 2007). Based on single nucleotide polymorphism, kompetitive allele-specific PCR (KASP) is a novel high-throughput genotyping technology, which possesses advantages such as high abundance, relative genetic stability, high accuracy and efficiency, convenience with low cost, and the ability to detect a large number of loci simultaneously (Semagn et al., 2014). This technology has already been applied to crops and ornamentals such as *Raphanus sativus* (Xing et al., 2024), *Brassica oleracea* (Shen et al., 2021), and *Cymbidium ensifolium* (Shen et al., 2025).

In recent years, studies have been conducted on the phenotypic traits, physiological and biochemical characteristics, and isozymes of *R. pseudoacacia*. Additionally, based on simple sequence repeat (SSR) markers, the genetic diversity and population structure characteristics of *R. pseudoacacia* populations have been revealed, laying a foundation for the genetic breeding and identification of this species (Guo et al., 2021; Yang et al., 2020; Szyp-Borowska et al., 2023; Mao et al., 2017). However, there is currently no research on the SNP-based DNA fingerprint database of *R. pseudoacacia*. Therefore, based on KASP technology, this study screened a set of SNP markers capable of distinguishing *R. pseudoacacia* germplasm resources, established DNA fingerprint profiles for 105 accessions of *R. pseudoacacia* and its closely related species, and effectively discriminated different elite germplasm or cultivars of *R. pseudoacacia*. This study provides a scientific basis and data reference for the genetic diversity analysis, cultivar identification, and molecular breeding of *R. pseudoacacia*.

## Materials and methods

### Plant materials and DNA extraction

**Figure 1:**
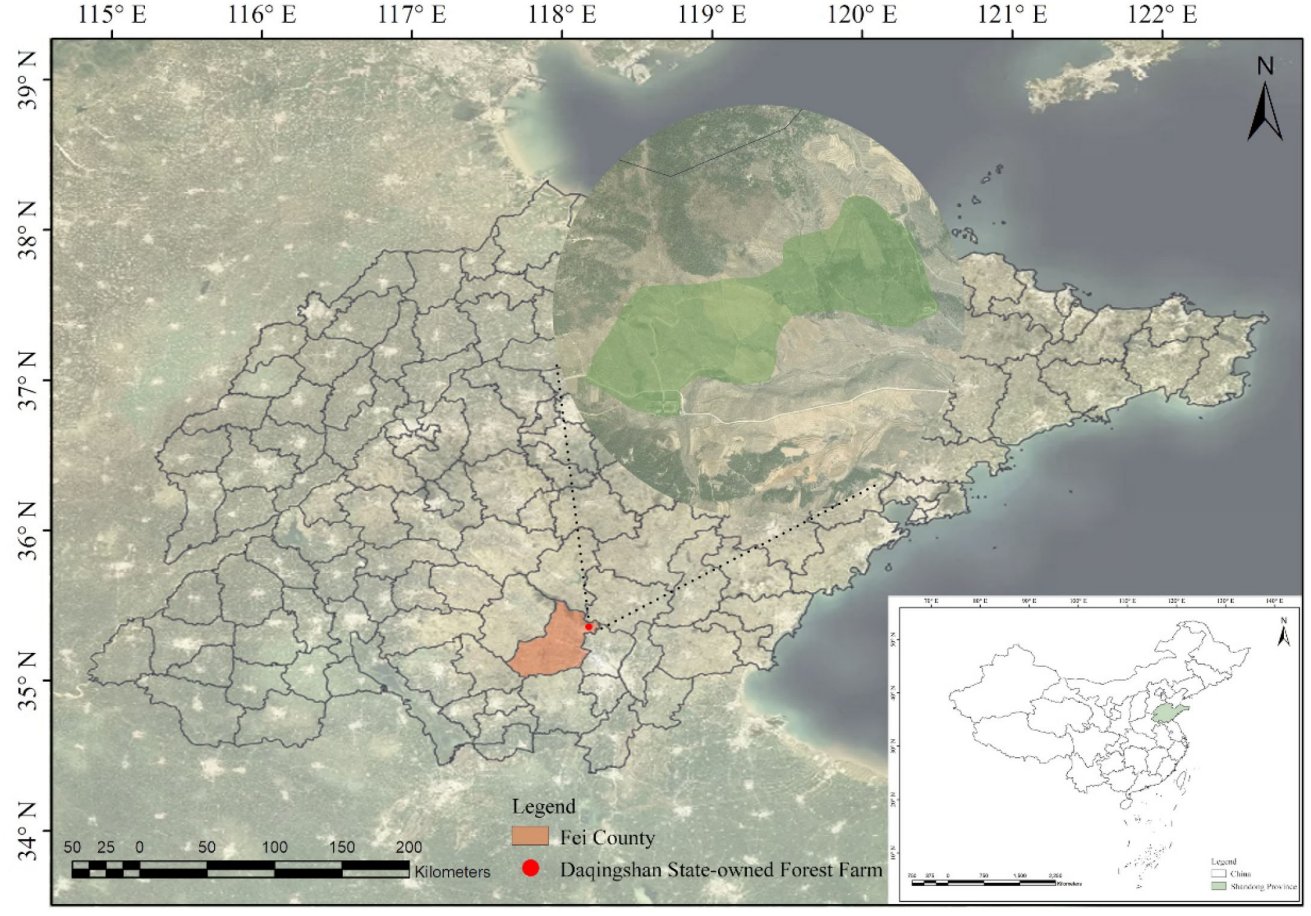
The geographic location of the sampling area in this study.

This study adhered to all relevant institutional, national, and international guidelines and laws. No prior permission was required to conduct research on this species. The materials used in this study were obtained from Daqingshan State-owned Forest Farm, Fei County, Shandong Province, China (**Figure 1**). DNA fingerprint profiles were constructed using 105 accessions of *R. pseudoacacia* and its closely related species, while 30 germplasm accessions of elite cultivar hybrid progenies of *R. pseudoacacia* were used for validating the fingerprint profiles (**Supplementary Table S1**).

DNA was extracted from the leaves via the cetyl-trimethylammonium bromide (CTAB) method (Porebski et al., 1997). Genomic DNA integrity was detected via agarose gel electrophoresis, and the DNA concentration and purity were determined via Nanodrop. The concentration of DNA detected by the Qubit 3.0 instrument was greater than 200 ng/μL. Samples were diluted with double-distilled water (ddH_2_O) to achieve a final concentration of 20–30 ng/μL.

### KASP development

VCFtools v.0.1.13 was used to filter the SNP loci obtained from our previous sequencing data (Wang et al., 2025; Danecek et al., 2011). To ensure that the selected SNP loci are characterized by distinctness, accuracy, and high polymorphism, four rounds of specificity screening were performed as follows: (1) retaining loci with a minor allele frequency (MAF) > 0.05; (2) removing loci with a missing rate > 0.2; (3) retaining loci with a polymorphism information content (PIC) value > 0.3; (4) retaining loci with no other variations within 100 bp upstream and downstream of the target locus.

Using the *R. pseudoacacia* genome as reference (Wang et al., 2023), 100 bp of flanking sequence on each side of every filtered SNP was extracted to design KASP primers. KASP primers were designed with the online tool SNPWay (http://www.snpway.com/). Among the successful assays, only those targeting SNPs distributed evenly across the chromosomes were retained for further use. Each KASP primer set consists of two forward primers (F_1_ and F_2_) and one reverse primer (R). The 5’ end of F_1_ carries the FAM tail sequence 5’-GAAGGTGACCAAGTTCATGCT-3’, whereas the 5’ end of F_2_ carries the HEX tail sequence 5’-GAAGGTCGGAGTCAACGGATT-3’.

### KASP genotyping

Primer mix was prepared by combining 12 µL of each forward primer (F_1_ and F_2_, 100μM), 30 µL of reverse primer (R, 100μM), and ddH_2_O to a final volume of 100 µL. PCR was performed in 96-well plates; each 10 µL reaction contained 2 µL DNA template, 0.14 µL primer mix, 5 µL 2 × KASP Master Mix (Beijing Jiacheng Biotechnology Co., Ltd), and 3 µL ddH_2_O. Two no-template controls (NTC) were included in every assay. The 2 × KASP Master Mix contains FAM and HEX fluorescent probes that can bind to the fluorescent tag sequences at the 5’ end of the primers. During PCR amplification, these probes recognize their corresponding fluorescent tag sequences, enabling the detection of fluorescent signals upon the completion of PCR and the subsequent determination of genotypes based on the endpoint fluorescence intensity.

A real-time fluorescence detection system was used for PCR and subsequent fluorescent signal collection. The thermal profile was: 95°C for 10 min (initial denaturation); 10 touch-down cycles of 95°C for 20 s, 61–55°C for 40 s (decreasing by 0.6°C per cycle); followed by 32 cycles of 95°C for 20 s, 55°C for 40 s. After the final cycle, fluorescence was read; if clusters remained unclear, additional cycles (95°C 20 s, 55°C 40 s) were run in sets of three until distinct genotypes were obtained.

### KASP screening and validation

Twenty *R. pseudoacacia* samples were randomly selected from the resequenced germplasm to validate the successfully designed KASP primers. Each sample was subjected to three technical replicates in independent PCR reactions to evaluate the repeatability of the assay. Among these, polymorphic primers with clear grouping, high genotyping accuracy, and good repeatability were selected as core primers. Based on the screened core primers, genotyping was performed on 105 accessions of *R. pseudoacacia* and its closely related species, and DNA fingerprint profiles were constructed. Thirty germplasm of elite cultivar hybrid progenies were used for the validation of both the core primers and the fingerprint profiles.

### Data analysis

Based on the genotyping data, genetic diversity parameters were calculated using PowerMarker v3.25, including minor allele frequency (MAF), polymorphism information content (PIC), heterozygosity, and gene diversity. Nei’s genetic-distance matrix was computed and used to construct a neighbor-joining (NJ) tree, which was visualized with MEGA 11. Population genetic structure was inferred using STRUCTURE 2.3.1 (Porras-Hurtado et al., 2013). The number of genetic clusters (K) was tested from 1 to 10. For each K, ten independent runs were performed under the admixture model, each with a burn-in of 10,000 steps followed by 100,000 MCMC iterations. After the runs, the optimal K was determined according to Evanno’s method (Evanno et al., 2005) using the online tool Structure Selector (https://lmme.ac.cn/StructureSelector). Principal component analysis (PCA) was performed using Tassel 5 (Bradbury et al., 2007). KASP genotyping results were visualized as a fingerprint heatmap in R: homozygous AA (yellow), GG (orange), CC (blue), TT (purple); heterozygous genotypes (green); missing data (grey).

## Results

### KASP development

**Figure 2:**
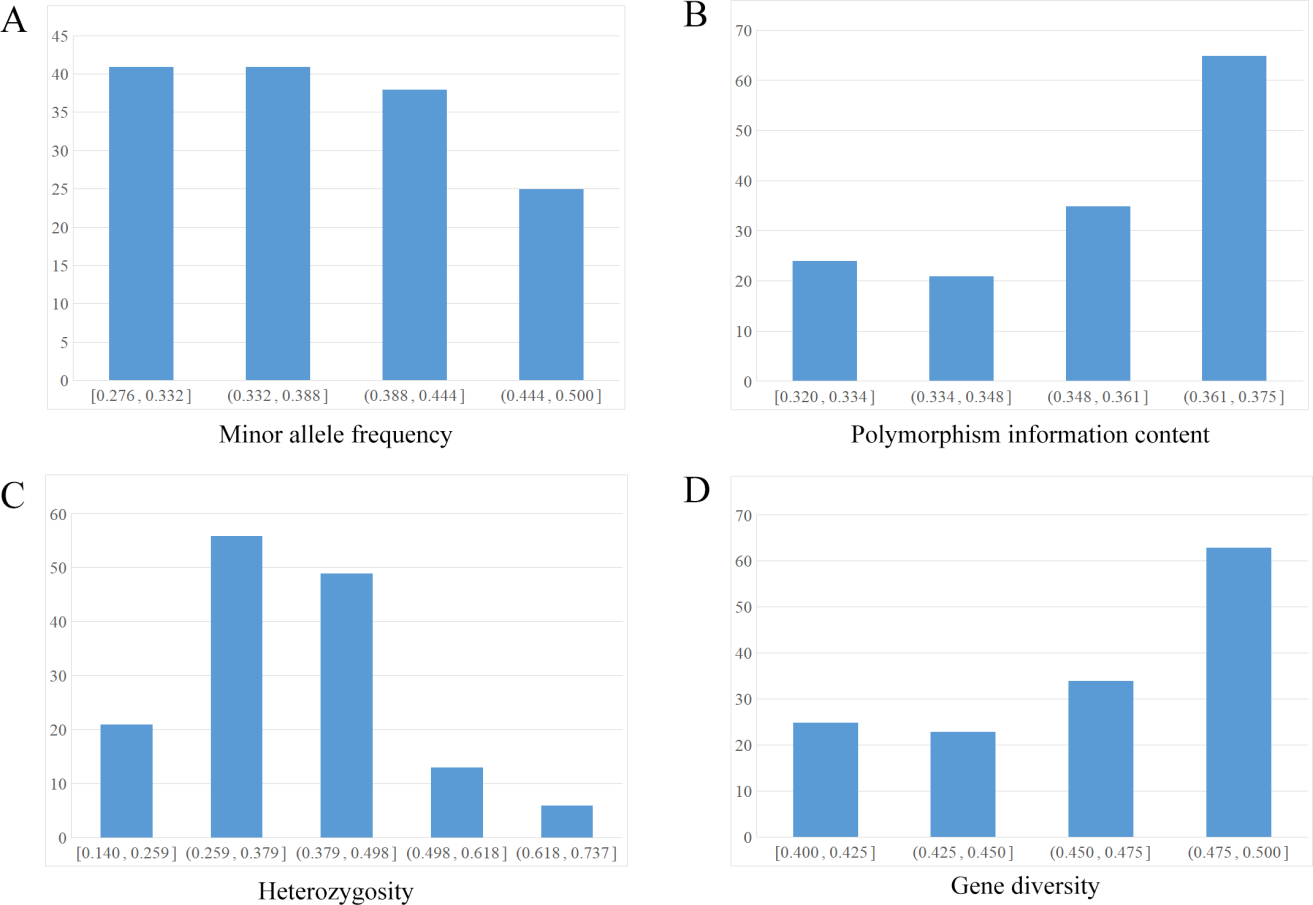
Distribution of MAF, PIC, heterozygosity and gene diversity indices for the 145 SNP loci. **(A)** Minor allele frequency (MAF). **(B)** Polymorphism information content (PIC). **(C)** Heterozygosity. **(D)** Gene diversity.

After screening, a total of 145 high-quality SNP loci were selected for subsequent KASP primer design. Genetic diversity parameters and distribution statistics were computed for these 145 SNP loci. The results showed that the minor allele frequency (MAF) and polymorphism information content (PIC) values ranged from 0.320 to 0.375 and 0.276 to 0.500, with mean values of 0.354 and 0.375, respectively (**Figures 2A, B**). Among these loci, 69.0% had a PIC value greater than 0.350. The average heterozygosity and average gene diversity were 0.378 (range: 0.140–0.737) and 0.461 (range: 0.400–0.500), respectively (**Figures 2C, D**).

### KASP screening

**Figure 3:**
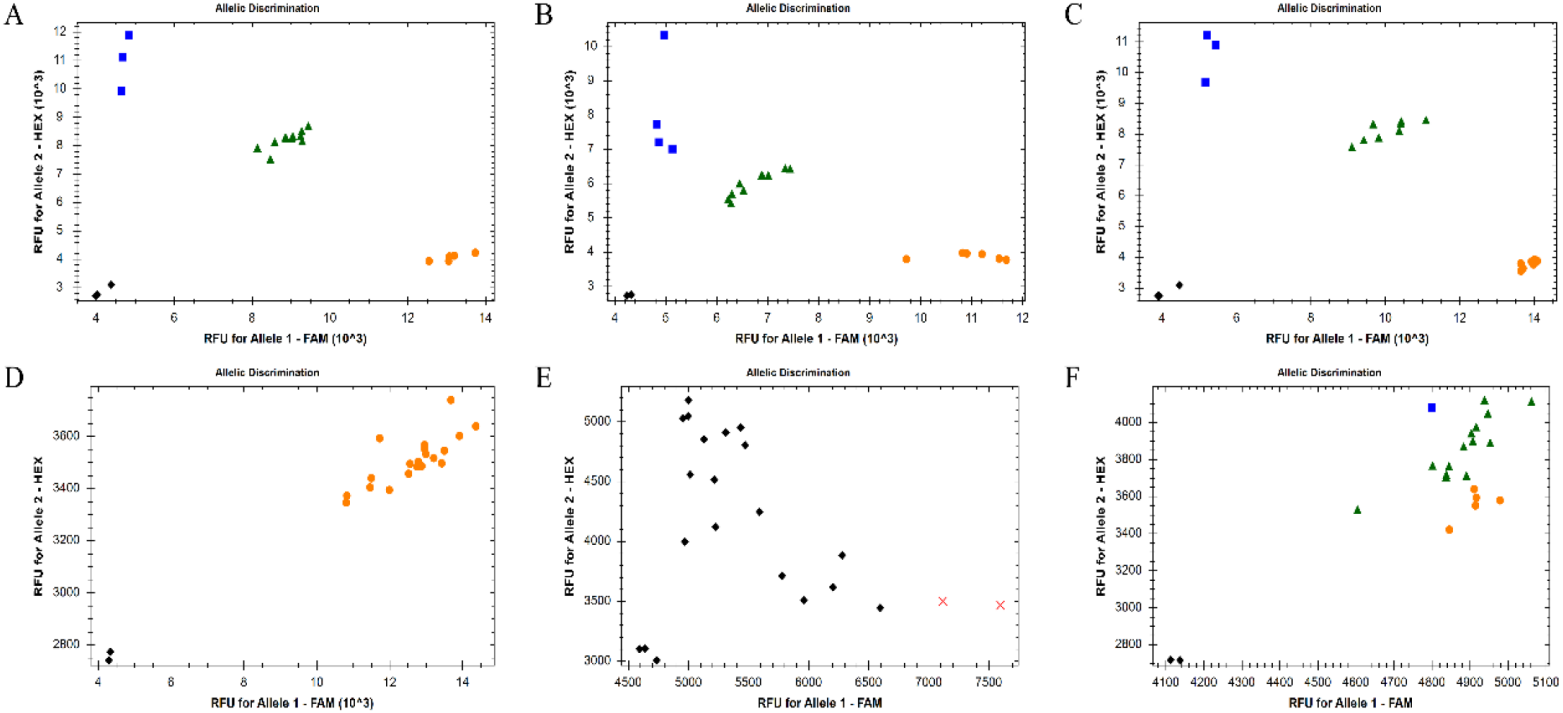
Representative KASP genotyping clusters for representative SNP loci. Orange dots and blue dots represent two different homozygous genotypes, respectively, while green dots represent heterozygous genotypes. Black dots and crosses indicate no fluorescent signal detected. **(A–C)** show the types retained during preliminary genotyping, and **(D–F)** show the types discarded during preliminary genotyping.

A total of 145 SNP loci were converted into KASP markers, among which KASP primers were successfully designed for 136 loci, resulting in a conversion success rate of 93.79%. From these 136 SNP loci, 65 loci with high polymorphism and uniform distribution on chromosomes were selected for KASP primer development. Genotyping was performed on 20 randomly selected *R. pseudoacacia* germplasm accessions to conduct preliminary screening of the developed primers. Representative results are shown in **Figure 3**.

The inter-assay consistency rate of the three replicate experiments remained above 97%, demonstrating the high reliability and repeatability of the KASP genotyping system. Among the 65 developed KASP markers, 31 exhibited clear genotype clusters and polymorphism with satisfactory genotyping results, which were selected as candidate core markers for subsequent analysis. 24 KASP markers failed to genotype successfully or showed indistinct genotyping results, while 10 KASP markers displayed only one genotype and lacked polymorphism. The genotyping error rate was calculated based on the inconsistent results of replicate experiments, and the genotyping error rates of all successfully genotyped primers were less than 5%. Detailed information of the developed KASP markers is provided in **Supplementary Table S2**.

### Genotyping results of the core KASP markers

All 105 accessions were successfully genotyped using the 31 KASP markers, and the genotyping results are provided in **Supplementary Table S3**. Genetic diversity analysis revealed (**Table 1**) that all 31 KASP markers exhibited favorable genotyping performance. Specifically, the polymorphism information content (PIC) values ranged from 0.245 to 0.375 with a mean of 0.335, while the minor allele frequency (MAF) values varied from 0.173 to 0.490 with an average of 0.328. The heterozygosity and gene diversity values ranged from 0.038 to 0.598 and 0.286 to 0.500, with mean values of 0.357 and 0.428, respectively. Among all 31 core KASP markers, the missing rates were all less than 5%, and 10 markers had no missing data. These results indicate that the 31 core KASP markers possess high genetic diversity and are suitable for genetic diversity analysis and identification of *R. pseudoacacia* germplasm resources.

**Table 1:** Genetic diversity information of 31 KASP markers.

Marker	MAF	Gene diversity	Heterozygosity	PIC	Missing rate
Rp0-1	0.385	0.473	0.423	0.361	0.95%
Rp0-4	0.490	0.500	0.392	0.375	2.86%
Rp1-2	0.352	0.456	0.419	0.352	0.00%
Rp1-4	0.248	0.373	0.438	0.303	0.00%
Rp1-6	0.360	0.461	0.380	0.355	4.76%
Rp2-1	0.282	0.405	0.097	0.323	1.90%
Rp2-2	0.252	0.377	0.390	0.306	0.00%
Rp2-6	0.361	0.461	0.067	0.355	0.95%
Rp3-1	0.319	0.435	0.238	0.340	0.00%
Rp3-3	0.356	0.458	0.385	0.353	0.95%
Rp4-1	0.199	0.319	0.340	0.268	1.90%
Rp4-3	0.296	0.417	0.282	0.330	1.90%
Rp4-5	0.411	0.484	0.525	0.367	3.81%
Rp5-1	0.422	0.488	0.495	0.369	1.90%
Rp5-3	0.252	0.377	0.406	0.306	3.81%
Rp5-4	0.450	0.495	0.426	0.373	3.81%
Rp6-2	0.284	0.407	0.333	0.324	2.86%
Rp6-3	0.173	0.286	0.038	0.245	0.95%
Rp6-4	0.271	0.396	0.086	0.317	0.00%
Rp7-1	0.443	0.493	0.105	0.372	0.00%
Rp7-2	0.214	0.337	0.371	0.280	0.00%
Rp7-3	0.436	0.492	0.598	0.371	2.86%
Rp7-5	0.421	0.487	0.584	0.369	3.81%
Rp8-1	0.229	0.353	0.400	0.290	0.00%
Rp8-8	0.320	0.435	0.380	0.341	4.76%
Rp8-9	0.381	0.472	0.465	0.360	3.81%
Rp9-1	0.311	0.428	0.427	0.337	1.90%
Rp9-4	0.422	0.488	0.495	0.369	1.90%
Rp9-5	0.314	0.431	0.400	0.338	0.00%
Rp10-5	0.300	0.420	0.390	0.332	0.00%
Rp10-8	0.228	0.352	0.277	0.290	3.81%
Mean	0.328	0.428	0.357	0.335	1.81%

### Population structure analysis

**Figure 4:**
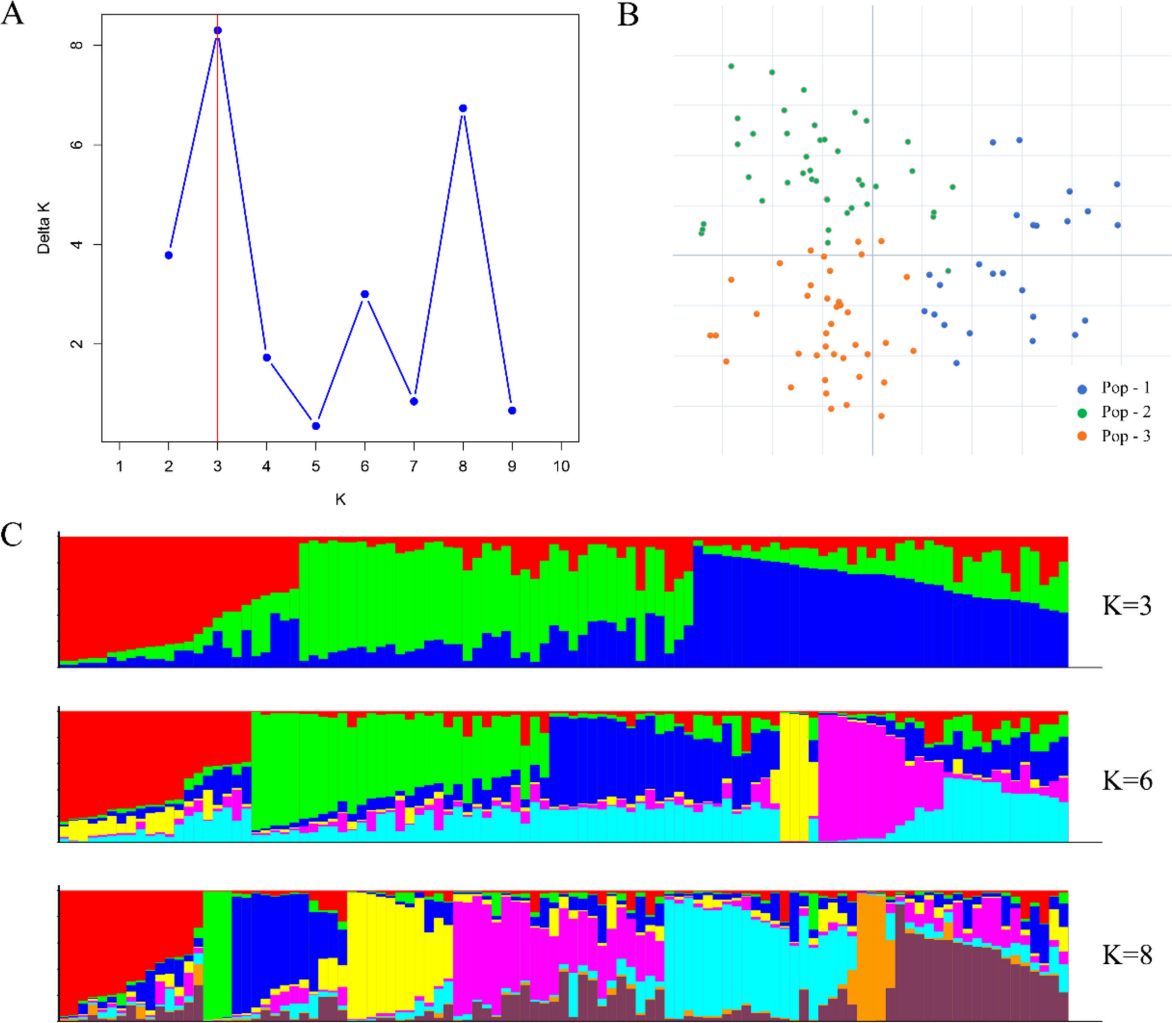
Genetic diversity analysis based on 31 KASP markers. **(A)** The Delta *K* values corresponding to different *K* measurements. **(B)** Principal component analysis. **(C)** Population structure at different values of *K*.

**Figure 5:**
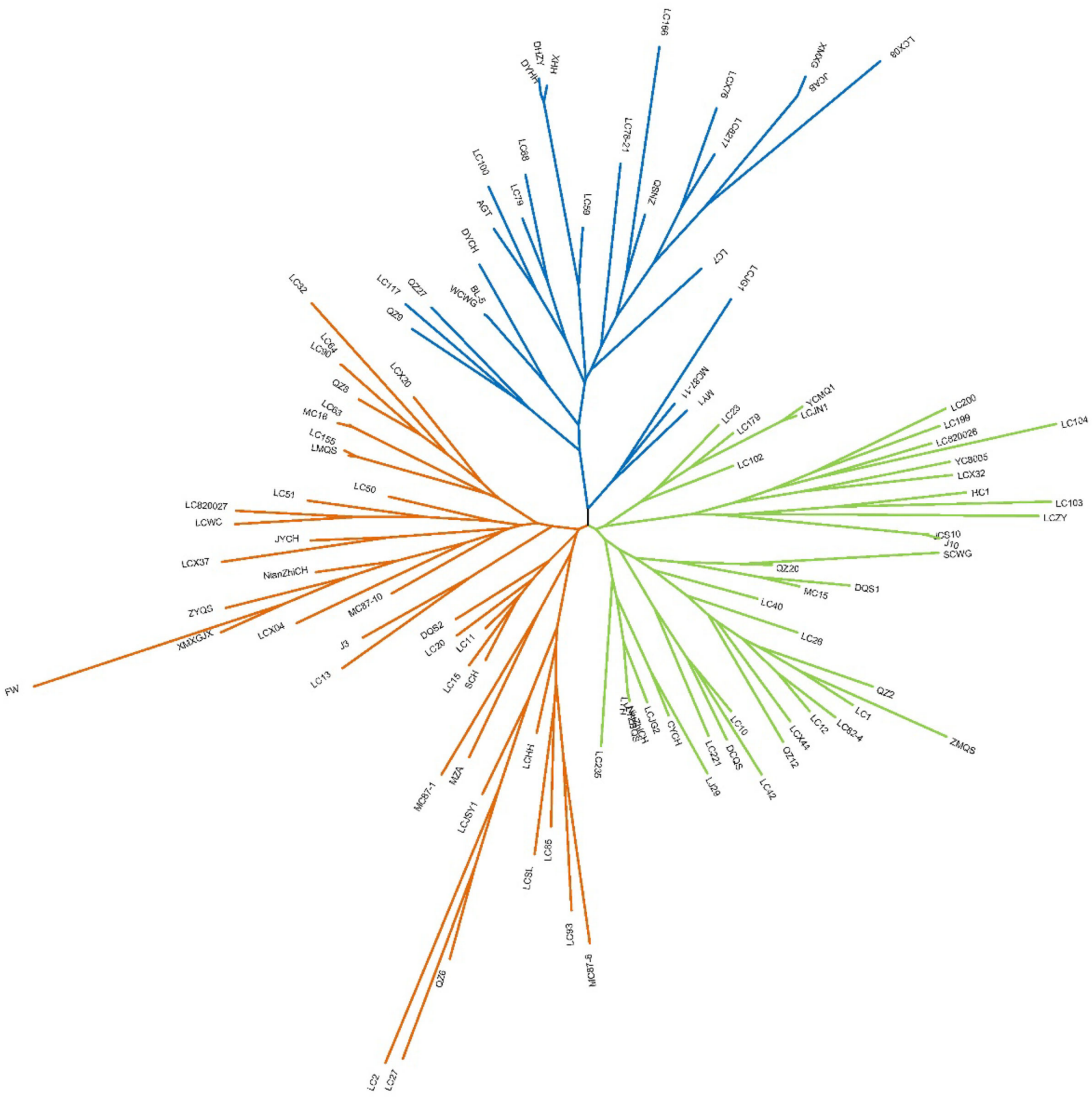
Phylogenetic tree constructed by the NJ method with 1000 bootstrap replicates. Orange: Pop-1; Blue: Pop-2; Green: Pop-3.

The Evanno analysis method has a limitation in that it cannot identify the scenario of *K* = 1. In the results of this study (**Figure 4A**), a distinct inflection point was observed for Delta *K*, which confirms the presence of population structure and thus excludes the possibility of *K* = 1. When *K* = 3, 6 and 8, Delta *K* showed peak inflection points in all cases; therefore, we inferred that the 105 germplasm accessions could be divided into 3, 6 or 8 groups (**Figure 4C**). In the Evanno analysis method, the *K* value corresponding to the highest peak of Delta *K* is generally prioritized, namely *K* = 3. Hence, in this study, the 105 samples used were tentatively divided into 3 subpopulations. In the genetic structure plot at *K* = 3, genetic component admixture was observed among the three subpopulations. Meanwhile, the genetic structure plots at *K* = 6 and *K* = 8 were also meaningful, as it could be seen that under these two *K* values, some individual subpopulations exhibited significantly different genetic components from other subpopulations. Principal Component Analysis (PCA) results showed that the 105 *R. pseudoacacia* and its closely related species clustered into three groups: Pop-1 (23.9%), Pop-2 (39.0%), and Pop-3 (37.1%) (**Figure 4B**). However, the differentiation among the three populations was not distinct, suggesting close genetic relationships between the germplasm accessions. Based on the generated genetic distance matrix (p-distance) (**Supplementary Table S4**), a phylogenetic tree was constructed using the neighbor-joining (NJ) method, which classified the 105 accessions into three clades: Pop-1 (39 accessions, orange), Pop-2 (26 accessions, blue), and Pop-3 (40 accessions, green) (**Figure 5**). Among these, three pairs of germplasm accessions (NiuZhiCH vs. LYZBQS, LC90 vs. LC64, and DHZY vs. DYHH) exhibited low genetic distances, indicating high genetic similarity. The consistency between the population structure analysis and cluster analysis validates the study results and enhances the reliability of the identified genetic populations.

### Fingerprint construction and validation

**Figure 6:**
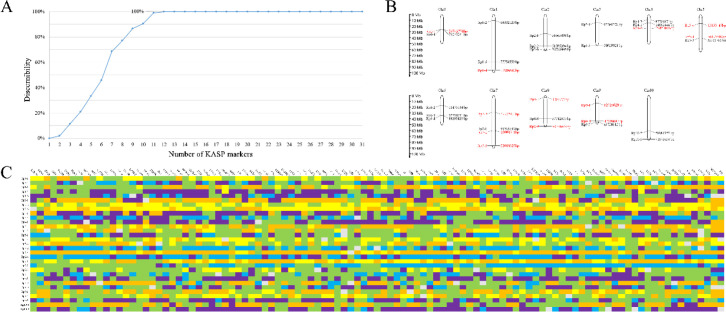
Fingerprinting analysis of 105 *R. pseudoacacia* and its closely related species germplasms. **(A)** Efficiency of combined SNP markers for identification. **(B)** Distribution of 31 KASP markers on *R. pseudoacacia* chromosomes. The red markers represent the 12 primers that can completely distinguish the 105 samples. **(C)** Fingerprinting of 105 *R. pseudoacacia* and its closely related species germplasms. Each row represents a genome, and each column represents a sample. Homozygous genotypes are shown in yellow (AA), orange (GG), blue (CC), and purple (TT); heterozygous genotypes are shown in green; and undetected genotypes are shown in gray.

With the increase in the number of primers, the identification efficiency of the 105 accessions gradually improved (**Figure 6A**). A complete discrimination of all 105 accessions was achieved using 12 primer pairs (Rp7-3, Rp7-5, Rp4-5, Rp5-1, Rp9-4, Rp8-9, Rp1-4, Rp9-1, Rp5-4, Rp0-1, Rp7-2, Rp8-1). The chromosomal distribution of the 31 KASP markers in *R. pseudoacacia* is illustrated in **Figure 6B**, where the 12 aforementioned primer pairs are highlighted in red.

DNA fingerprint profiles of the 105 accessions were constructed based on the 31 KASP markers. In the fingerprint profiles (**Figure 6C**), each row represents one SNP genotype, and each column corresponds to one accession. The genotypes AA, GG, CC, and TT are denoted by yellow, orange, blue, and purple, respectively; heterozygous genotypes are indicated in green, while missing data are shown in gray. Fingerprint profile analysis revealed that three pairs of accessions (NiuZhiCH vs. LYZBQS, LC90 vs. LC64, and DHZY vs. DYHH) differed at only one locus, exhibiting high genetic similarity, which is consistent with the results of the cluster analysis.

Thirty unsequenced samples were genotyped using the 31 core KASP markers, and the results were compared with the previously constructed DNA fingerprint (**Supplementary Table S5**). Genotyping results showed that all 30 samples exhibited varying degrees of differences from the germplasm accessions in the fingerprint database, with no exact matches to any existing cultivars. The overall identification rate of the fingerprint database for new germplasm reached 100%. The 31 KASP markers developed in this study exhibited reliable discriminatory ability, providing technical support and a theoretical basis for the analysis of genetic relationships, germplasm collection, and conservation of *R. pseudoacacia* and its related species in Daqingshan Forest Farm.

## Discussion

As breeding objectives for forest trees become more diverse and technology advances, *R. pseudoacacia* improvement has shifted from single-trait to multi-trait selection. Integrating conventional breeding with molecular tools now allows breeders to evaluate and enhance genetic diversity more efficiently, shortening breeding cycles, increasing the precision of elite-germplasm selection, and reducing the overall cost of genetic improvement (McGregor et al., 2000). This continual increase in *R. pseudoacacia* germplasm has led to obvious homogeneity, hampering both accurate identification and efficient conservation. An effective identification system is therefore urgently needed for cultivar authentication and germplasm management. Most earlier studies of *R. pseudoacacia* employed RAPD or SSR markers; for example, Shu et al. used randomly amplified polymorphic DNA (RAPD) to detect somaclonal variation among shoots regenerated from axillary buds of tetraploid *R. pseudoacacia* (Shu et al., 2003). Guo et al. developed and applied SSR markers to assess genetic variation among different provenances of *R. pseudoacacia* (Guo et al., 2017, 2018). In contrast, SNP-based KASP assays are cheaper, more stable, and considerably faster than gel-based PCR markers, and they are now widely used for germplasm identification, marker-assisted breeding, gene mapping, and seed-purity testing (Grewal et al., 2020; Rasheed et al., 2016; Liu et al., 2018). In *Ipomoea batatas* (Yang et al., 2024), *Vitis vinifera* (Wang F. et al., 2022) and *Nicotiana tabacum* (Wang et al., 2021), core sets of 74, 25 and 47 KASP markers, respectively, have already been defined for cultivar discrimination and fingerprint construction, illustrating the broad utility of this platform for germplasm management and breeding support.

In this study, the obtained SNP loci were subjected to four rounds of specificity screening. The filtering parameters were referenced to the criteria described by Diao et al. (2024), while the screening threshold for PIC was elevated in our study. A more stringent screening threshold contributes to improved efficiency in core marker screening for the obtained loci. Finally, 31 core SNP loci were selected and converted into KASP markers, which were further applied to genetic structure analysis and DNA fingerprinting construction. To ensure these markers provide reliable estimates of diversity and accurate locus identification, they must be derived from high-quality SNPs (Bus et al., 2012). The 31 KASP markers exhibited mean MAF and PIC values of 0.328 and 0.335, respectively—levels comparable to those reported for *Raphanus sativus* (Xing et al., 2024) (0.303 and 0.320) and *Brassica oleracea* (Shen et al., 2021) (0.330 and 0.330). Thus, the core KASP markers developed in this study possess high polymorphism and stability.

Genetic structure describes how genetic variation is distributed within a population. It reflects the population’s genetic makeup and internal relationships, revealing the interplay of evolutionary history, adaptive change, breeding system, gene flow, and natural selection that the lineage has experienced over time (Huang et al., 2021). STRUCTURE analysis with the 31 KASP markers split the 105 accessions into three sub-groups that nevertheless remained closely related (mean Nei’s distance = 0.2706, indicating high overall similarity). Notably, XHH–DYHH–DHZY, LYH–LYZBQS and LC64–LC90 showed almost identical genotypes, suggesting they are near-identical lots.

A DNA fingerprint is a profile generated with molecular markers that directly reveals the genetic differences among individuals at the DNA level (Sucher et al., 2012). Molecular markers are extremely numerous, highly polymorphic, and widely distributed across the genome; they can be scored at any developmental stage and can distinguish homozygous from heterozygous genotypes in cultivars or breeding lines, offering powerful tools for genetic improvement (Nybom et al., 2014). Consequently, DNA fingerprinting is widely used to assess genetic relationships among crop varieties, to verify seed purity, and for many other applications in plant research (Yang et al., 2022; Wang L. et al., 2022; Satturu et al., 2018). In this study, 31 core KASP markers were used to build a fingerprint of 105 accessions; only 12 of these markers were sufficient for complete discrimination. Three pairs—NiuZhiCH vs. LYZBQS, LC90 vs. LC64, and DHZY vs. DYHH—differed at a single locus, confirming the genetic-structure analysis. Additionally, 30 non-sequenced accessions were genotyped with the 31 core markers; all profiles differed to varying degrees from every reference cultivar and could not be matched to any entry, confirming the high inter-cultivar resolution and intra-cultivar stability of SNP. With the advancement of breeding technologies, these markers have the potential to improve the accuracy and efficiency of *R. pseudoacacia* cultivar identification, and may lay a preliminary theoretical foundation for new-cultivar protection. They could also serve as a promising preliminary step toward optimizing the distinctness, uniformity and stability (DUS) testing standard for *R. pseudoacacia*. However, it should be noted that the above prospects are based on the current results obtained from a limited set of germplasm accessions under specific experimental conditions. Further systematic validation is required before the markers can be formally integrated into DUS testing protocols and new-cultivar protection practices. Specifically, additional validation work should include: (1) multi-environment testing across different ecological regions of *R. pseudoacacia* cultivation to verify the stability of marker amplification and polymorphism under varying climatic and soil conditions; (2) multi-generation verification of the core markers in successive generations of the same cultivar to confirm their consistency in long-term application; and (3) comparative analysis with traditional morphological DUS testing to evaluate the complementarity of molecular markers and phenotypic indicators, thereby improving the comprehensiveness of cultivar identification systems.

## Conclusions

Based on the previously sequenced genome data of *R. pseudoacacia*, SNP markers were developed following strict screening criteria in this study and further integrated with KASP technology to select 31 core SNPs. These core SNPs were applied to analyze the genetic diversity and genetic structure of 105 *R. pseudoacacia* individuals and their related species in Daqingshan Forest Farm, while also constructing DNA fingerprinting profiles for these germplasm accessions. The selected marker combination exhibited good polymorphism and high efficiency, which could completely distinguish all 105 germplasm accessions. The KASP rapid detection system targeting the core SNPs of *R. pseudoacacia* in this study has the advantages of strong discriminatory ability, convenient operation and low cost, and could serve as a promising preliminary attempt to optimize the DUS testing standard system for *R. pseudoacacia*.

## Data Availability

The original contributions presented in the study are included in the article/**Supplementary Material**. Further inquiries can be directed to the corresponding authors.
