# Supplementary material for: Development of KASP markers, SNP fingerprinting and population structure analysis of Robinia pseudoacacia and its closely related species
Source: Front Plant Sci. 2026 Feb 3;17:1761477. doi: 10.3389/fpls.2026.1761477 (PMC12909524; doi:10.3389/fpls.2026.1761477)
Supplement: Supplementary file 1 [file Table1.docx]

**Table S1** Detailed information of the germplasm resources used.

| No. | Sample name | Provenance | Sampling date | Geographic coordinates | Species | Background | Comments |
| --- | --- | --- | --- | --- | --- | --- | --- |
| 1 | DQS1 | China | 2024.5.17 | 35.390278741°N, 118.200562132°E | *Robinia pseudoacacia* | Artificially bred cultivar | It has been sequenced and used for preliminary primer screening |
| 2 | DQS2 | China | 2024.5.17 | 35.389410372°N, 118.199875421°E | *Robinia pseudoacacia* | Artificially bred cultivar | It has been sequenced and used for preliminary primer screening |
| 3 | DCQS | China | 2024.5.17 | 35.391865293°N, 118.20234871°E | *Robinia pseudoacacia* | Artificially bred cultivar | It has been sequenced and used for preliminary primer screening |
| 4 | LMQS | China | 2024.5.18 | 35.388954614°N, 118.197893563°E | *Robinia pseudoacacia* | Artificially bred cultivar | It has been sequenced |
| 5 | ZMQS | China | 2024.5.17 | 35.392103555°N, 118.203987244°E | *Robinia pseudoacacia* | Artificially bred cultivar | It has been sequenced and used for preliminary primer screening |
| 6 | ZYQS | China | 2024.5.17 | 35.390876226°N, 118.201156895°E | *Robinia pseudoacacia* | Artificially bred cultivar | It has been sequenced and used for preliminary primer screening |
| 7 | HC1 | China | 2024.5.17 | 35.389987457°N, 118.198562376°E | *Robinia pseudoacacia* | Artificially bred cultivar | It has been sequenced and used for preliminary primer screening |
| 8 | MY1 | China | 2024.5.17 | 35.391543288°N, 118.204123587°E | *Robinia pseudoacacia* | Artificially bred cultivar | It has been sequenced and used for preliminary primer screening |
| 9 | QSNZ | China | 2024.5.17 | 35.388765329°N, 118.199235718°E | *Robinia pseudoacacia* | Artificially bred cultivar | It has been sequenced |
| 10 | LYZBQS | China | 2024.5.18 | 35.39254319°N, 118.200987659°E | *Robinia pseudoacacia* | Bud mutation of 'LYH' | It has been sequenced |
| 11 | MZA | America | 2024.5.18 | 35.389123451°N, 118.201876542°E | *Robinia pseudoacacia* | Introduced asexual system | It has been sequenced |
| 12 | AGT | Argentina | 2024.5.18 | 35.390567893°N, 118.197234567°E | *Robinia pseudoacacia* | Introduced asexual system | It has been sequenced and used for preliminary primer screening |
| 13 | XHH | Spain | 2024.5.18 | 35.391234564°N, 118.203456789°E | *Robinia pseudoacacia* | Introduced cross breed | It has been sequenced |
| 14 | DHZY | Spain | 2024.5.17 | 35.388654325°N, 118.198987654°E | *Robinia pseudoacacia* | Family of ’XHH‘ | It has been sequenced |
| 15 | CYCH | America | 2024.5.17 | 35.392345676°N, 118.202123456°E | *Robinia pseudoacacia* | Variety of cultivated | It has been sequenced and used for preliminary primer screening |
| 16 | LC1 | China | 2024.5.18 | 35.389789017°N, 118.19954321°E | *Robinia pseudoacacia* | Selected superior asexual system | It has been sequenced and used for preliminary primer screening |
| 17 | LC10 | China | 2024.5.18 | 35.390321458°N, 118.200123457°E | *Robinia pseudoacacia* | Selected superior asexual system | It has been sequenced and used for preliminary primer screening |
| 18 | LC100 | China | 2024.5.17 | 35.391987659°N, 118.204567898°E | *Robinia pseudoacacia* | Selected superior asexual system | It has been sequenced and used for preliminary primer screening |
| 19 | LC102 | China | 2024.5.17 | 35.38854321°N, 118.197654321°E | *Robinia pseudoacacia* | Selected superior asexual system | It has been sequenced |
| 20 | LC103 | China | 2024.5.17 | 35.392123451°N, 118.203123452°E | *Robinia pseudoacacia* | Selected superior asexual system | It has been sequenced |
| 21 | LC104 | China | 2024.5.17 | 35.389987653°N, 118.199234563°E | *Robinia pseudoacacia* | Selected superior asexual system | It has been sequenced |
| 22 | LC11 | China | 2024.5.17 | 35.390765434°N, 118.200876544°E | *Robinia pseudoacacia* | Selected superior asexual system | It has been sequenced |
| 23 | LC117 | China | 2024.5.17 | 35.391432105°N, 118.202654325°E | *Robinia pseudoacacia* | Selected superior asexual system | It has been sequenced |
| 24 | LC12 | China | 2024.5.17 | 35.388876546°N, 118.198345676°E | *Robinia pseudoacacia* | Selected superior asexual system | It has been sequenced |
| 25 | LC13 | China | 2024.5.17 | 35.392456787°N, 118.203987657°E | *Robinia pseudoacacia* | Selected superior asexual system | It has been sequenced |
| 26 | LC15 | China | 2024.5.17 | 35.389321458°N, 118.199012348°E | *Robinia pseudoacacia* | Selected superior asexual system | It has been sequenced |
| 27 | LC155 | China | 2024.5.17 | 35.390123459°N, 118.200432109°E | *Robinia pseudoacacia* | Artificially bred cultivar | It has been sequenced |
| 28 | LC166 | China | 2024.5.17 | 35.39165432°N, 118.20423456°E | *Robinia pseudoacacia* | Selected superior asexual system | It has been sequenced |
| 29 | LC179 | China | 2024.5.17 | 35.388432101°N, 118.197987651°E | *Robinia pseudoacacia* | Selected superior asexual system | It has been sequenced |
| 30 | LC199 | China | 2024.5.17 | 35.392234562°N, 118.203543212°E | *Robinia pseudoacacia* | Selected superior asexual system | It has been sequenced and used for preliminary primer screening |
| 31 | LC2 | China | 2024.5.17 | 35.389654323°N, 118.199765433°E | *Robinia pseudoacacia* | Selected superior asexual system | It has been sequenced |
| 32 | LC20 | China | 2024.5.17 | 35.390987654°N, 118.201234564°E | *Robinia pseudoacacia* | Selected superior asexual system | It has been sequenced |
| 33 | LC200 | China | 2024.5.17 | 35.391321455°N, 118.202987655°E | *Robinia pseudoacacia* | Selected superior asexual system | It has been sequenced |
| 34 | LC221 | China | 2024.5.17 | 35.388765436°N, 118.198654326°E | *Robinia pseudoacacia* | Selected superior asexual system | It has been sequenced |
| 35 | LC23 | China | 2024.5.17 | 35.392567897°N, 118.204432107°E | *Robinia pseudoacacia* | Selected superior asexual system | It has been sequenced |
| 36 | LC235 | China | 2024.5.18 | 35.389012348°N, 118.199345678°E | *Robinia pseudoacacia* | Selected superior asexual system | It has been sequenced |
| 37 | LC26 | China | 2024.5.17 | 35.390432109°N, 118.200765439°E | *Robinia pseudoacacia* | Selected superior asexual system | It has been sequenced |
| 38 | LC27 | China | 2024.5.17 | 35.39187654°N, 118.20323456°E | *Robinia pseudoacacia* | Selected superior asexual system | It has been sequenced |
| 39 | LC32 | China | 2024.5.17 | 35.388567891°N, 118.198012341°E | *Robinia pseudoacacia* | Selected superior asexual system | It has been sequenced |
| 40 | LC40 | China | 2024.5.17 | 35.392012342°N, 118.203765432°E | *Robinia pseudoacacia* | Selected superior asexual system | It has been sequenced |
| 41 | LC42 | China | 2024.5.17 | 35.389876543°N, 118.199987653°E | *Robinia pseudoacacia* | Selected superior asexual system | It has been sequenced |
| 42 | LC50 | China | 2024.5.17 | 35.390654324°N, 118.201432104°E | *Robinia pseudoacacia* | Selected superior asexual system | It has been sequenced |
| 43 | LC51 | China | 2024.5.17 | 35.391543215°N, 118.203543215°E | *Robinia pseudoacacia* | Selected superior asexual system | It has been sequenced |
| 44 | LC59 | China | 2024.5.17 | 35.388654326°N, 118.198345676°E | *Robinia pseudoacacia* | Selected superior asexual system | It has been sequenced |
| 45 | LC63 | China | 2024.5.17 | 35.392345677°N, 118.204123457°E | *Robinia pseudoacacia* | Selected superior asexual system | It has been sequenced |
| 46 | LC64 | China | 2024.5.17 | 35.389234568°N, 118.199654328°E | *Robinia pseudoacacia* | Selected superior asexual system | It has been sequenced |
| 47 | LC68 | China | 2024.5.17 | 35.390234569°N, 118.200987659°E | *Robinia pseudoacacia* | Selected superior asexual system | It has been sequenced |
| 48 | LC7 | China | 2024.5.17 | 35.39176543°N, 118.20387654°E | *Robinia pseudoacacia* | Selected superior asexual system | It has been sequenced |
| 49 | LC78-21 | China | 2024.5.17 | 35.388432101°N, 118.197765432°E | *Robinia pseudoacacia* | Selected superior asexual system | It has been sequenced and used for preliminary primer screening |
| 50 | LC79 | China | 2024.5.17 | 35.392098762°N, 118.203345673°E | *Robinia pseudoacacia* | Selected superior asexual system | It has been sequenced |
| 51 | LC820026 | China | 2024.5.17 | 35.389543213°N, 118.199123454°E | *Robinia pseudoacacia* | Excellent types of breeding | It has been sequenced |
| 52 | LC820027 | China | 2024.5.17 | 35.390789015°N, 118.201567895°E | *Robinia pseudoacacia* | Excellent types of breeding | It has been sequenced |
| 53 | LC8217 | China | 2024.5.17 | 35.391345676°N, 118.202789016°E | *Robinia pseudoacacia* | Selected superior asexual system | It has been sequenced |
| 54 | LC82-4 | China | 2024.5.18 | 35.388890127°N, 118.198876547°E | *Robinia pseudoacacia* | Selected superior asexual system | It has been sequenced |
| 55 | LC85 | China | 2024.5.17 | 35.392467898°N, 118.204012348°E | *Robinia pseudoacacia* | Selected superior asexual system | It has been sequenced |
| 56 | LC90 | China | 2024.5.17 | 35.389345679°N, 118.199456789°E | *Robinia pseudoacacia* | Selected superior asexual system | It has been sequenced |
| 57 | LC93 | China | 2024.5.17 | 35.39014567°N, 118.20056789°E | *Robinia pseudoacacia* | Selected superior asexual system | It has been sequenced |
| 58 | LCHH | China | 2024.5.17 | 35.391678901°N, 118.204345671°E | *Robinia pseudoacacia* | Selected superior asexual system | It has been sequenced |
| 59 | LCJG1 | China | 2024.5.17 | 35.388578902°N, 118.197890122°E | *Robinia pseudoacacia* | Selected superior asexual system | It has been sequenced and used for preliminary primer screening |
| 60 | LCJG2 | China | 2024.5.18 | 35.392245673°N, 118.203678903°E | *Robinia pseudoacacia* | Selected superior asexual system | It has been sequenced |
| 61 | LCJSY1 | China | 2024.5.17 | 35.389765434°N, 118.199890124°E | *Robinia pseudoacacia* | Selected superior asexual system | It has been sequenced |
| 62 | LCJN1 | China | 2024.5.17 | 35.390901235°N, 118.201345675°E | *Robinia pseudoacacia* | Selected superior asexual system | It has been sequenced |
| 63 | LCSL | China | 2024.5.17 | 35.391256786°N, 118.202890126°E | *Robinia pseudoacacia* | Selected superior asexual system | It has been sequenced and used for preliminary primer screening |
| 64 | LCWC | China | 2024.5.18 | 35.388789017°N, 118.198765437°E | *Robinia pseudoacacia* | Selected superior asexual system | It has been sequenced |
| 65 | LCZY | China | 2024.5.18 | 35.392578908°N, 118.204567898°E | *Robinia pseudoacacia* | Selected superior asexual system | It has been sequenced |
| 66 | LJ29 | China | 2024.5.17 | 35.389023459°N, 118.199567899°E | *Robinia pseudoacacia* | Selected superior asexual system | It has been sequenced |
| 67 | BL-5 | China | 2024.5.17 | 35.39044567°N, 118.20087654°E | *Robinia pseudoacacia* | Selected superior asexual system | It has been sequenced |
| 68 | J10 | China | 2024.5.17 | 35.391887651°N, 118.203345671°E | *Robinia pseudoacacia* | Selected superior asexual system | It has been sequenced |
| 69 | J3 | China | 2024.5.17 | 35.388589012°N, 118.198123452°E | *Robinia pseudoacacia* | Selected superior asexual system | It has been sequenced |
| 70 | JCAB | China | 2024.5.17 | 35.392023453°N, 118.203876543°E | *Robinia pseudoacacia* | variety | It has been sequenced |
| 71 | JCS10 | China | 2024.5.17 | 35.389887654°N, 118.200012344°E | *Robinia pseudoacacia* | Selected superior asexual system | It has been sequenced |
| 72 | LCX04 | China | 2024.5.18 | 35.390665435°N, 118.201543215°E | *Robinia pseudoacacia* | Selected superior asexual system | It has been sequenced |
| 73 | LCX08 | China | 2024.5.18 | 35.391554326°N, 118.203654326°E | *Robinia pseudoacacia* | Selected superior asexual system | It has been sequenced and used for preliminary primer screening |
| 74 | LCX20 | China | 2024.5.18 | 35.388665437°N, 118.198456787°E | *Robinia pseudoacacia* | Selected superior asexual system | It has been sequenced |
| 75 | LCX32 | China | 2024.5.17 | 35.392356788°N, 118.204234568°E | *Robinia pseudoacacia* | Selected superior asexual system | It has been sequenced |
| 76 | LCX37 | China | 2024.5.17 | 35.389245679°N, 118.199765439°E | *Robinia pseudoacacia* | Selected superior asexual system | It has been sequenced |
| 77 | LCX44 | China | 2024.5.17 | 35.39024567°N, 118.20109876°E | *Robinia pseudoacacia* | Selected superior asexual system | It has been sequenced |
| 78 | LCX76 | China | 2024.5.17 | 35.391776541°N, 118.203987651°E | *Robinia pseudoacacia* | Selected superior asexual system | It has been sequenced and used for preliminary primer screening |
| 79 | YC8005 | China | 2024.5.17 | 35.388443212°N, 118.197876542°E | *Robinia pseudoacacia* | Selected superior asexual system | It has been sequenced |
| 80 | YCMQ1 | China | 2024.5.17 | 35.392109873°N, 118.203456783°E | *Robinia pseudoacacia* | Selected superior asexual system | It has been sequenced |
| 81 | QZ12 | China | 2024.5.17 | 35.389554324°N, 118.199234564°E | *Robinia pseudoacacia* | Full-sib family (LC10×LC48*) | It has been sequenced |
| 82 | QZ20 | China | 2024.5.17 | 35.390790125°N, 118.201678905°E | *Robinia pseudoacacia* | Full-sib family (LC13*×LC9*) | It has been sequenced |
| 83 | QZ27 | China | 2024.5.17 | 35.391356786°N, 118.202890126°E | *Robinia pseudoacacia* | Full-sib family (LC2×LC155*) | It has been sequenced |
| 84 | QZ2 | China | 2024.5.17 | 35.388891237°N, 118.198987657°E | *Robinia pseudoacacia* | Full-sib family (LC10×LC1) | It has been sequenced |
| 85 | QZ6 | China | 2024.5.17 | 35.392478908°N, 118.204123458°E | *Robinia pseudoacacia* | Full-sib family (LC2×LC155) | It has been sequenced |
| 86 | QZ8 | China | 2024.5.18 | 35.389356789°N, 118.199567899°E | *Robinia pseudoacacia* | Full-sib family (LC64×LC102*) | It has been sequenced |
| 87 | QZ9 | China | 2024.5.17 | 35.39015678°N, 118.2006789°E | *Robinia pseudoacacia* | Full-sib family (XMXG×LC102) | It has been sequenced |
| 88 | MC15 | America | 2024.5.17 | 35.391689011°N, 118.204456781°E | *Robinia pseudoacacia* | Introduced provenance | It has been sequenced and used for preliminary primer screening |
| 89 | MC16 | America | 2024.5.17 | 35.388589012°N, 118.197901232°E | *Robinia pseudoacacia* | Introduced provenance | It has been sequenced |
| 90 | MC87-1 | America | 2024.5.17 | 35.392256783°N, 118.203789013°E | *Robinia pseudoacacia* | Family of introduced provenance | It has been sequenced |
| 91 | MC87-10 | America | 2024.5.17 | 35.389776544°N, 118.199901234°E | *Robinia pseudoacacia* | Family of introduced provenance | It has been sequenced |
| 92 | MC87-11 | America | 2024.5.17 | 35.390912345°N, 118.201456785°E | *Robinia pseudoacacia* | Family of introduced provenance | It has been sequenced |
| 93 | MC87-8 | America | 2024.5.17 | 35.391267896°N, 118.202901236°E | *Robinia pseudoacacia* | Family of introduced provenance | It has been sequenced and used for preliminary primer screening |
| 94 | NiuZhiCH | America | 2024.5.17 | 35.388790127°N, 118.198876547°E | *Robinia pseudoacacia* | Artificially bred forma | It has been sequenced |
| 95 | LYH | America | 2024.5.18 | 35.392589018°N, 118.204678908°E | *Robinia pseudoacacia* | Artificially bred forma | It has been sequenced |
| 96 | SCH | America | 2024.5.18 | 35.389034569°N, 118.199678909°E | *Robinia pseudoacacia* | Forma of transgene | It has been sequenced |
| 97 | DYHH | America | 2024.5.17 | 35.39045678°N, 118.20098765°E | *Robinia pseudoacacia* | Progeny variation of ' XHH' | It has been sequenced |
| 98 | JYCH | America | 2024.5.18 | 35.391898761°N, 118.203456781°E | *Robinia pseudoacacia* | Progeny variation of black locust | It has been sequenced |
| 99 | DYCH | America | 2024.5.18 | 35.388590122°N, 118.198234562°E | *Robinia pseudoacacia* | Progeny variation of black locust | It has been sequenced |
| 100 | SCWG | China | 2024.5.18 | 35.392034563°N, 118.203987653°E | *Robinia pseudoacacia* | Selected superior asexual system | It has been sequenced |
| 101 | WCWG | China | 2024.5.18 | 35.389898764°N, 118.200123454°E | *Robinia pseudoacacia* | Selected superior asexual system | It has been sequenced |
| 102 | NianZhiCH | America | 2024.5.17 | 35.390676545°N, 118.201654325°E | *Robinia* *viscose* | Introduced asexual system | It has been sequenced |
| 103 | XMXG | Mexico | 2024.5.17 | 35.391565436°N, 118.203765436°E | *Robinia* *Nuo-Mexicana* | Introduced variety | It has been sequenced |
| 104 | XMXGJX | Mexico | 2024.5.18 | 35.388676547°N, 118.198567897°E | *Robinia Nuo-Mexicana* | Introduced half-sib families | It has been sequenced |
| 105 | FW | America | 2024.5.18 | 35.392367898°N, 118.204345678°E | *Robinia* *hispida* | Introduced half-sib families | It has been sequenced |
| 106 | 133×MQ2 | China | 2024.7.30 | 35.389997152°N, 118.196428521°E | *Robinia pseudoacacia* | Hybrid progeny of improved varieties | It has not been sequenced and is used for core primer validation |
| 107 | 152×6815-2 | China | 2024.7.30 | 35.389563213°N, 118.195987342°E | *Robinia pseudoacacia* | Hybrid progeny of improved varieties | It has not been sequenced and is used for core primer validation |
| 108 | 133×MQ2 15-5 | China | 2024.7.30 | 35.390421874°N, 118.197234193°E | *Robinia pseudoacacia* | Hybrid progeny of improved varieties | It has not been sequenced and is used for core primer validation |
| 109 | 15-63 | China | 2024.7.30 | 35.389210545°N, 118.194987654°E | *Robinia pseudoacacia* | Hybrid progeny of improved varieties | It has not been sequenced and is used for core primer validation |
| 110 | 07×X25 15-1 | China | 2024.7.30 | 35.390187656°N, 118.196876545°E | *Robinia pseudoacacia* | Hybrid progeny of improved varieties | It has not been sequenced and is used for core primer validation |
| 111 | X1×42 15-1 | China | 2024.7.30 | 35.389789017°N, 118.195654326°E | *Robinia pseudoacacia* | Hybrid progeny of improved varieties | It has not been sequenced and is used for core primer validation |
| 112 | 133×DH 15-4 | China | 2024.7.30 | 35.390345678°N, 118.197012347°E | *Robinia pseudoacacia* | Hybrid progeny of improved varieties | It has not been sequenced and is used for core primer validation |
| 113 | 229×J4 15-10 | China | 2024.7.30 | 35.389103459°N, 118.195234568°E | *Robinia pseudoacacia* | Hybrid progeny of improved varieties | It has not been sequenced and is used for core primer validation |
| 114 | HC 15-25 | China | 2024.7.30 | 35.39001234°N, 118.196654329°E | *Robinia pseudoacacia* | Hybrid progeny of improved varieties | It has not been sequenced and is used for core primer validation |
| 115 | 133×DH 15-5 | China | 2024.7.30 | 35.389654321°N, 118.19589012°E | *Robinia pseudoacacia* | Hybrid progeny of improved varieties | It has not been sequenced and is used for core primer validation |
| 116 | 57×X15 15-24 | China | 2024.7.30 | 35.390234562°N, 118.196987651°E | *Robinia pseudoacacia* | Hybrid progeny of improved varieties | It has not been sequenced and is used for core primer validation |
| 117 | 57×X15 15-39 | China | 2024.7.30 | 35.389345673°N, 118.195123452°E | *Robinia pseudoacacia* | Hybrid progeny of improved varieties | It has not been sequenced and is used for core primer validation |
| 118 | 57×X15 15-9 | China | 2024.7.30 | 35.389876544°N, 118.196234563°E | *Robinia pseudoacacia* | Hybrid progeny of improved varieties | It has not been sequenced and is used for core primer validation |
| 119 | 29J4 15-21 | China | 2024.7.30 | 35.390512345°N, 118.197345674°E | *Robinia pseudoacacia* | Hybrid progeny of improved varieties | It has not been sequenced and is used for core primer validation |
| 120 | 152×62 15-17 | China | 2024.7.30 | 35.389098766°N, 118.194876545°E | *Robinia pseudoacacia* | Hybrid progeny of improved varieties | It has not been sequenced and is used for core primer validation |
| 121 | X46 GC 15-1 | China | 2024.7.30 | 35.390456787°N, 118.197123456°E | *Robinia pseudoacacia* | Hybrid progeny of improved varieties | It has not been sequenced and is used for core primer validation |
| 122 | DH42 15-01 | China | 2024.7.30 | 35.389234568°N, 118.195345677°E | *Robinia pseudoacacia* | Hybrid progeny of improved varieties | It has not been sequenced and is used for core primer validation |
| 123 | 152×J4 15-1 | China | 2024.7.30 | 35.389901239°N, 118.196543218°E | *Robinia pseudoacacia* | Hybrid progeny of improved varieties | It has not been sequenced and is used for core primer validation |
| 124 | 152×10 15-4 | China | 2024.7.30 | 35.39012345°N, 118.196765439°E | *Robinia pseudoacacia* | Hybrid progeny of improved varieties | It has not been sequenced and is used for core primer validation |
| 125 | 152×10 15-21 | China | 2024.7.30 | 35.389543211°N, 118.19578901°E | *Robinia pseudoacacia* | Hybrid progeny of improved varieties | It has not been sequenced and is used for core primer validation |
| 126 | 152×J4 15-3 | China | 2024.7.30 | 35.389765432°N, 118.196012341°E | *Robinia pseudoacacia* | Hybrid progeny of improved varieties | It has not been sequenced and is used for core primer validation |
| 127 | 80-102×02ZC 15-1 | China | 2024.7.30 | 35.390321093°N, 118.197210982°E | *Robinia pseudoacacia* | Hybrid progeny of improved varieties | It has not been sequenced and is used for core primer validation |
| 128 | 13 ZC 05-1 | China | 2024.7.30 | 35.389123454°N, 118.195012343°E | *Robinia pseudoacacia* | Hybrid progeny of improved varieties | It has not been sequenced and is used for core primer validation |
| 129 | 07ZC 05-03 | China | 2024.7.30 | 35.390098765°N, 118.196812344°E | *Robinia pseudoacacia* | Hybrid progeny of improved varieties | It has not been sequenced and is used for core primer validation |
| 130 | HC 15-3 | China | 2024.7.30 | 35.389678906°N, 118.195912345°E | *Robinia pseudoacacia* | Hybrid progeny of improved varieties | It has not been sequenced and is used for core primer validation |
| 131 | X9 | China | 2024.7.30 | 35.390210987°N, 118.196912346°E | *Robinia pseudoacacia* | Hybrid progeny of improved varieties | It has not been sequenced and is used for core primer validation |
| 132 | 42×DH 15-1 | China | 2024.7.30 | 35.389321098°N, 118.195210987°E | *Robinia pseudoacacia* | Hybrid progeny of improved varieties | It has not been sequenced and is used for core primer validation |
| 133 | 42×DH 15-2 | China | 2024.7.30 | 35.389890129°N, 118.196321098°E | *Robinia pseudoacacia* | Hybrid progeny of improved varieties | It has not been sequenced and is used for core primer validation |
| 134 | 80-10×01 ZC 1501 | China | 2024.7.30 | 35.39054321°N, 118.197432109°E | *Robinia pseudoacacia* | Hybrid progeny of improved varieties | It has not been sequenced and is used for core primer validation |
| 135 | X5 | China | 2024.7.30 | 35.389087651°N, 118.19491234°E | *Robinia pseudoacacia* | Hybrid progeny of improved varieties | It has not been sequenced and is used for core primer validation |

*LC48, LC13, LC9, LC155, LC102: All above are selected superior asexual system. But they are not used as samples in this study.
