# Supplementary material for: Development of KASP markers, SNP fingerprinting and population structure analysis of Robinia pseudoacacia and its closely related species
Source: Front Plant Sci. 2026 Feb 3;17:1761477. doi: 10.3389/fpls.2026.1761477 (PMC12909524; doi:10.3389/fpls.2026.1761477)
Supplement: Supplementary file 2 [file Table2.docx]

**Table S2** KASP primer information and preliminary screening genotyping results.

| **Primer No.** | **Primer Name** | **Chromosome** | **Variation Position** | **Variation Type** | **Primer** | **Genotyping Results** |
| --- | --- | --- | --- | --- | --- | --- |
| Rp_0_24743774 | Rp0-1 | 0 | 24743774 | A/G | F1: GAAGGTGACCAAGTTCATGCTTTTCCTTGCCTATTGTTGCTTTCA | Success |
|  |  |  |  |  | F2: GAAGGTCGGAGTCAACGGATTTTTCCTTGCCTATTGTTGCTTTCG |  |
|  |  |  |  |  | R: TTCAAACACCCATGTAAAGGAAGC |  |
| Rp_0_35831671 | Rp0-2 | 0 | 35831671 | T/C | F1: GAAGGTGACCAAGTTCATGCTCCAAAAAGGTAAAATTAAGTGCTGAACT | Ambiguous |
|  |  |  |  |  | F2: GAAGGTCGGAGTCAACGGATTCCAAAAAGGTAAAATTAAGTGCTGAACC |  |
|  |  |  |  |  | R: CACTGCTTTGGGAAAGTTAGAAGG |  |
| Rp_0_46310282 | Rp0-3 | 0 | 46310282 | C/A | F1: GAAGGTGACCAAGTTCATGCTTTAGTGTTGGCTCTTGATTTGGAC | Ambiguous |
|  |  |  |  |  | F2: GAAGGTCGGAGTCAACGGATTTTAGTGTTGGCTCTTGATTTGGAA |  |
|  |  |  |  |  | R: GCAGGCAAGTGGTAAGTTAGATCT |  |
| Rp_0_25073091 | Rp0-4 | 0 | 25073091 | T/C | F1: GAAGGTGACCAAGTTCATGCTTGATGAGTGTTGTTGACATGAAATT | Success |
|  |  |  |  |  | F2: GAAGGTCGGAGTCAACGGATTTGATGAGTGTTGTTGACATGAAATC |  |
|  |  |  |  |  | R: CTAAGCCCATGGTCTGGTTATCTT |  |
| Rp_0_26562659 | Rp0-5 | 0 | 26562659 | T/A | F1: GAAGGTGACCAAGTTCATGCTCCTTCTCAAATACCACGCCTCTT | No Polymorphism |
|  |  |  |  |  | F2: GAAGGTCGGAGTCAACGGATTCCTTCTCAAATACCACGCCTCTA |  |
|  |  |  |  |  | R: ATCAAGAGGATGAGGATGTGTCAC |  |
| Rp_1_1854617 | Rp1-1 | 1 | 1854617 | C/A | F1: GAAGGTGACCAAGTTCATGCTCCTCAGATATGCGTATATTCACATGTC | Ambiguous |
|  |  |  |  |  | F2: GAAGGTCGGAGTCAACGGATTCCTCAGATATGCGTATATTCACATGTA |  |
|  |  |  |  |  | R: ATTTACTCGGAGAAAGAGCCTAGG |  |
| Rp_1_10092129 | Rp1-2 | 1 | 10092129 | G/A | F1: GAAGGTGACCAAGTTCATGCTAGATGACTTCTATCAGAAAACTGTCG | Success |
|  |  |  |  |  | F2: GAAGGTCGGAGTCAACGGATTAGATGACTTCTATCAGAAAACTGTCA |  |
|  |  |  |  |  | R: ATGCAGCAGTCATTCTAGAGTCAT |  |
| Rp_1_72048494 | Rp1-3 | 1 | 72048494 | G/T | F1: GAAGGTGACCAAGTTCATGCTCGACCTCCAACATGATGTCCG | No Polymorphism |
|  |  |  |  |  | F2: GAAGGTCGGAGTCAACGGATTCGACCTCCAACATGATGTCCT |  |
|  |  |  |  |  | R: GAAATGTTTGACGGAAGAAGAGGG |  |
| Rp_1_91986640 | Rp1-4 | 1 | 91986640 | T/A | F1: GAAGGTGACCAAGTTCATGCTAAAATTTGGCCTTGCTAGTGTTTT | Success |
|  |  |  |  |  | F2: GAAGGTCGGAGTCAACGGATTAAAATTTGGCCTTGCTAGTGTTTA |  |
|  |  |  |  |  | R: ATTAGCAAAAACCGGTACTGCAAA |  |
| Rp_1_24908042 | Rp1-5 | 1 | 24908042 | G/T | F1: GAAGGTGACCAAGTTCATGCTTGTATAACTCAAGTTAAGCATGAAGAAG | Ambiguous |
|  |  |  |  |  | F2: GAAGGTCGGAGTCAACGGATTTGTATAACTCAAGTTAAGCATGAAGAAT |  |
|  |  |  |  |  | R: AGAGATGCAGTACCAGTTTTTGTT |  |
| Rp_1_77754558 | Rp1-6 | 1 | 77754558 | T/C | F1: GAAGGTGACCAAGTTCATGCTGAATCTGGCCTCACCTCTTGCT | Success |
|  |  |  |  |  | F2: GAAGGTCGGAGTCAACGGATTGAATCTGGCCTCACCTCTTGCC |  |
|  |  |  |  |  | R: AAAACAAGAGGGTTTCAATGCCAA |  |
| Rp_2_31665898 | Rp2-1 | 2 | 31665898 | G/T | F1: GAAGGTGACCAAGTTCATGCTCGTTAATTTGTGTTGGTTGGTTTCG | Success |
|  |  |  |  |  | F2: GAAGGTCGGAGTCAACGGATTCGTTAATTTGTGTTGGTTGGTTTCT |  |
|  |  |  |  |  | R: ACCGATCACAAAATCAGAAACACA |  |
| Rp_2_51595394 | Rp2-2 | 2 | 51595394 | A/G | F1: GAAGGTGACCAAGTTCATGCTTGCATGGCTGATTTGTTATCACAA | Success |
|  |  |  |  |  | F2: GAAGGTCGGAGTCAACGGATTTGCATGGCTGATTTGTTATCACAG |  |
|  |  |  |  |  | R: GAATTGCAATGGCTGTCCTATCTC |  |
| Rp_2_192605 | Rp2-3 | 2 | 192605 | A/T | F1: GAAGGTGACCAAGTTCATGCTATGAAGATTTTGTGGTGGGCAGAA | No Polymorphism |
|  |  |  |  |  | F2: GAAGGTCGGAGTCAACGGATTATGAAGATTTTGTGGTGGGCAGAT |  |
|  |  |  |  |  | R: CCAACACATGACATCCCAAAATCA |  |
| Rp_2_49939445 | Rp2-4 | 2 | 49939445 | C/T | F1: GAAGGTGACCAAGTTCATGCTTCATGAGTACTTTGATAATAAACAACCATC | Ambiguous |
|  |  |  |  |  | F2: GAAGGTCGGAGTCAACGGATTTCATGAGTACTTTGATAATAAACAACCATT |  |
|  |  |  |  |  | R: GATTTTTCCTCACTGGGTTGTCTG |  |
| Rp_2_4444452 | Rp2-5 | 2 | 4444452 | T/G | F1: GAAGGTGACCAAGTTCATGCTCCGCATATTTATTTTGTTTTACGCCT | No Polymorphism |
|  |  |  |  |  | F2: GAAGGTCGGAGTCAACGGATTCCGCATATTTATTTTGTTTTACGCCG |  |
|  |  |  |  |  | R: CTTTACTTGCTCTGCTATTCTGCC |  |
| Rp_2_58788005 | Rp2-6 | 2 | 58788005 | A/G | F1: GAAGGTGACCAAGTTCATGCTGGTAGAATTCCACCGAATTAGTACA | Success |
|  |  |  |  |  | F2: GAAGGTCGGAGTCAACGGATTGGTAGAATTCCACCGAATTAGTACG |  |
|  |  |  |  |  | R: CGTTGTTCCACCGAATTTGAAAAC |  |
| Rp_3_13201378 | Rp3-1 | 3 | 13201378 | T/G | F1: GAAGGTGACCAAGTTCATGCTAAACCTAAACAAACCGGCCGATA | Success |
|  |  |  |  |  | F2: GAAGGTCGGAGTCAACGGATTAAACCTAAACAAACCGGCCGATC |  |
|  |  |  |  |  | R: TGACAATAAAGGTCACAACTGGAT |  |
| Rp_3_50929657 | Rp3-2 | 3 | 50929657 | C/T | F1: GAAGGTGACCAAGTTCATGCTTCTATCTTAATCACATAAAAGATATCATTGC | Ambiguous |
|  |  |  |  |  | F2: GAAGGTCGGAGTCAACGGATTTCTATCTTAATCACATAAAAGATATCATTGT |  |
|  |  |  |  |  | R: AAACCATTCATCTTCAGGCAGTTC |  |
| Rp_3_50929921 | Rp3-3 | 3 | 50929921 | C/T | F1: GAAGGTGACCAAGTTCATGCTGCACAGTTTTATCCCTGTAAATTAGAAC | Success |
|  |  |  |  |  | F2: GAAGGTCGGAGTCAACGGATTGCACAGTTTTATCCCTGTAAATTAGAAT |  |
|  |  |  |  |  | R: ATTCGATTGAAGGGTAGGGAACAA |  |
| Rp_3_6655588 | Rp3-4 | 3 | 6655588 | A/C | F1: GAAGGTGACCAAGTTCATGCTACCGGATTAATAACCATAGAATTAGAAAT | No Polymorphism |
|  |  |  |  |  | F2: GAAGGTCGGAGTCAACGGATTACCGGATTAATAACCATAGAATTAGAAAG |  |
|  |  |  |  |  | R: CAATTCGCTATTGAAAGCCCTTCT |  |
| Rp_3_13179294 | Rp3-5 | 3 | 13179294 | C/T | F1: GAAGGTGACCAAGTTCATGCTGTCAAACCTAAACAAACCGGCC | Ambiguous |
|  |  |  |  |  | F2: GAAGGTCGGAGTCAACGGATTGTCAAACCTAAACAAACCGGCT |  |
|  |  |  |  |  | R: ACTGGATAATGCGAAAAGTTTTGGT |  |
| Rp_4_14062444 | Rp4-1 | 4 | 14062444 | T/G | F1: GAAGGTGACCAAGTTCATGCTGCCATGCAAAGGAGTCGCTT | Success |
|  |  |  |  |  | F2: GAAGGTCGGAGTCAACGGATTGCCATGCAAAGGAGTCGCTG |  |
|  |  |  |  |  | R: CCGGTTTAACCATGTCATCATCAC |  |
| Rp_4_14062620 | Rp4-2 | 4 | 14062620 | C/A | F1: GAAGGTGACCAAGTTCATGCTGTGTGATGTGGTAATTGGAACAGC | Ambiguous |
|  |  |  |  |  | F2: GAAGGTCGGAGTCAACGGATTGTGTGATGTGGTAATTGGAACAGA |  |
|  |  |  |  |  | R: TGTCCACACTTGCCATACATATCA |  |
| Rp_4_8770487 | Rp4-3 | 4 | 8770487 | G/A | F1: GAAGGTGACCAAGTTCATGCTGATTCGTTTGGTCACCGATTCG | Success |
|  |  |  |  |  | F2: GAAGGTCGGAGTCAACGGATTGATTCGTTTGGTCACCGATTCA |  |
|  |  |  |  |  | R: ATCAAACTCAGATGAAACTCGCAC |  |
| Rp_4_46285701 | Rp4-4 | 4 | 46285701 | G/A | F1: GAAGGTGACCAAGTTCATGCTTGGTATAACAGAATTGATTCTCACCTG | No Polymorphism |
|  |  |  |  |  | F2: GAAGGTCGGAGTCAACGGATTTGGTATAACAGAATTGATTCTCACCTA |  |
|  |  |  |  |  | R: AATCTTCTCATTTTGTGTCAGCGG |  |
| Rp_4_23451076 | Rp4-5 | 4 | 23451076 | G/A | F1: GAAGGTGACCAAGTTCATGCTCCACATCAAGGAACATCAAATTAAAAGTAC | Success |
|  |  |  |  |  | F2: GAAGGTCGGAGTCAACGGATTCCACATCAAGGAACATCAAATTAAAAGTAT |  |
|  |  |  |  |  | R: GTGACTATGTTGAGACGTGTTTGG |  |
| Rp_5_36805502 | Rp5-1 | 5 | 36805502 | T/C | F1: GAAGGTGACCAAGTTCATGCTCCCTTCCCCTTCTTTGCACT | Success |
|  |  |  |  |  | F2: GAAGGTCGGAGTCAACGGATTCCCTTCCCCTTCTTTGCACC |  |
|  |  |  |  |  | R: ACAAAAGATGGGTGCTTTGAAAGA |  |
| Rp_5_38697223 | Rp5-2 | 5 | 38697223 | A/G | F1: GAAGGTGACCAAGTTCATGCTAATCCATCATTTTCATCACAGCTAA | Ambiguous |
|  |  |  |  |  | F2: GAAGGTCGGAGTCAACGGATTAATCCATCATTTTCATCACAGCTAG |  |
|  |  |  |  |  | R: AACAGGAGGAGCTGTAAATCGTAG |  |
| Rp_5_38935765 | Rp5-3 | 5 | 38935765 | A/G | F1: GAAGGTGACCAAGTTCATGCTAGTTCGATTTGCTTAATCAGTATTGAA | Success |
|  |  |  |  |  | F2: GAAGGTCGGAGTCAACGGATTAGTTCGATTTGCTTAATCAGTATTGAG |  |
|  |  |  |  |  | R: TATATGTGGGCAAAGGTGTTCTCA |  |
| Rp_5_15159147 | Rp5-4 | 5 | 15159147 | G/A | F1: GAAGGTGACCAAGTTCATGCTTGCAAGTAGCTAAGCTTTATACCTAG | Success |
|  |  |  |  |  | F2: GAAGGTCGGAGTCAACGGATTTGCAAGTAGCTAAGCTTTATACCTAA |  |
|  |  |  |  |  | R: AGGGACGGATCTAGGAATCAACTA |  |
| Rp_5_18487962 | Rp5-5 | 5 | 18487962 | G/A | F1: GAAGGTGACCAAGTTCATGCTTCATGGCTTTAGTGGATAAGATTAAATC | Ambiguous |
|  |  |  |  |  | F2: GAAGGTCGGAGTCAACGGATTTCATGGCTTTAGTGGATAAGATTAAATT |  |
|  |  |  |  |  | R: AACACTTGAACTAATGCATGCCAA |  |
| Rp_6_7056120 | Rp6-1 | 6 | 7056120 | G/A | F1: GAAGGTGACCAAGTTCATGCTTGGAATTTTGCTGAACACGAATTAC | Ambiguous |
|  |  |  |  |  | F2: GAAGGTCGGAGTCAACGGATTTGGAATTTTGCTGAACACGAATTAT |  |
|  |  |  |  |  | R: TTGTTCAACCGAATAACAACCTCG |  |
| Rp_6_16475934 | Rp6-2 | 6 | 16475934 | C/T | F1: GAAGGTGACCAAGTTCATGCTTGGTGGTATGACAATTATCGATGC | Success |
|  |  |  |  |  | F2: GAAGGTCGGAGTCAACGGATTTGGTGGTATGACAATTATCGATGT |  |
|  |  |  |  |  | R: GTACCCAACTAGAACAGCCAAAAC |  |
| Rp_6_32754651 | Rp6-3 | 6 | 32754651 | G/A | F1: GAAGGTGACCAAGTTCATGCTTGGTATCATTTTCTAACTGAAATTGCG | Success |
|  |  |  |  |  | F2: GAAGGTCGGAGTCAACGGATTTGGTATCATTTTCTAACTGAAATTGCA |  |
|  |  |  |  |  | R: GGTGTTAGTTCGCTGTTTTCTTCA |  |
| Rp_6_33235428 | Rp6-4 | 6 | 33235428 | C/T | F1: GAAGGTGACCAAGTTCATGCTAGCGAAATCCTCTCATTCGTAGC | Success |
|  |  |  |  |  | F2: GAAGGTCGGAGTCAACGGATTAGCGAAATCCTCTCATTCGTAGT |  |
|  |  |  |  |  | R: TGATGATGAATCACAGATTCGGAA |  |
| Rp_6_48095365 | Rp6-5 | 6 | 48095365 | A/G | F1: GAAGGTGACCAAGTTCATGCTGCCTTATAAACAAAGTAGCCACCA | Ambiguous |
|  |  |  |  |  | F2: GAAGGTCGGAGTCAACGGATTGCCTTATAAACAAAGTAGCCACCG |  |
|  |  |  |  |  | R: TTGCTCACATTAGTCACGGAATTG |  |
| Rp_7_55265643 | Rp7-1 | 7 | 55265643 | A/G | F1: GAAGGTGACCAAGTTCATGCTGGAAAGAATGAAGGAATGGAATTGGA | Success |
|  |  |  |  |  | F2: GAAGGTCGGAGTCAACGGATTGGAAAGAATGAAGGAATGGAATTGGG |  |
|  |  |  |  |  | R: CCTCTTCGCTATCCAATCCTCTTT |  |
| Rp_7_58008516 | Rp7-2 | 7 | 58008516 | C/T | F1: GAAGGTGACCAAGTTCATGCTCTCTCCAATGCTCTCTGTGAGG | Success |
|  |  |  |  |  | F2: GAAGGTCGGAGTCAACGGATTCTCTCCAATGCTCTCTGTGAGA |  |
|  |  |  |  |  | R: ACTCAGGTATGCAAATTCTTGTCT |  |
| Rp_7_27772901 | Rp7-3 | 7 | 27772901 | A/T | F1: GAAGGTGACCAAGTTCATGCTCGAGAATCTCACTCCTAAGATTGCT | Success |
|  |  |  |  |  | F2: GAAGGTCGGAGTCAACGGATTCGAGAATCTCACTCCTAAGATTGCA |  |
|  |  |  |  |  | R: CAATTCCAGTGCTCAAATGTGTCT |  |
| Rp_7_74311795 | Rp7-4 | 7 | 74311795 | T/C | F1: GAAGGTGACCAAGTTCATGCTTGAATGTTTGTAAAAATTGATTTTGAAGTGA | Unsuccessful |
|  |  |  |  |  | F2: GAAGGTCGGAGTCAACGGATTTGAATGTTTGTAAAAATTGATTTTGAAGTGG |  |
|  |  |  |  |  | R: AACATGTTGCTTTTGATTTTGAGTT |  |
| Rp_7_83018029 | Rp7-5 | 7 | 83018029 | C/T | F1: GAAGGTGACCAAGTTCATGCTGACATGCTTGTTCGGTCGAAG | Success |
|  |  |  |  |  | F2: GAAGGTCGGAGTCAACGGATTGACATGCTTGTTCGGTCGAAA |  |
|  |  |  |  |  | R: TAACCAGTGCAGTACCATCAGAAA |  |
| Rp_8_3365777 | Rp8-1 | 8 | 3365777 | T/C | F1: GAAGGTGACCAAGTTCATGCTCCACATGGATGGAAAAATGCAAAA | Success |
|  |  |  |  |  | F2: GAAGGTCGGAGTCAACGGATTCCACATGGATGGAAAAATGCAAAG |  |
|  |  |  |  |  | R: CATGACTGATGGAGAGGTGATGAA |  |
| Rp_8_11461166 | Rp8-2 | 8 | 11461166 | A/G | F1: GAAGGTGACCAAGTTCATGCTGTATGTGGAGCGTTCAATTGTGT | Ambiguous |
|  |  |  |  |  | F2: GAAGGTCGGAGTCAACGGATTGTATGTGGAGCGTTCAATTGTGC |  |
|  |  |  |  |  | R: TTTCGTGAGAGATTTGTCAATGTT |  |
| Rp_8_13017839 | Rp8-3 | 8 | 13017839 | C/T | F1: GAAGGTGACCAAGTTCATGCTACGGACCAAGAGACAATATTCCAG | Ambiguous |
|  |  |  |  |  | F2: GAAGGTCGGAGTCAACGGATTACGGACCAAGAGACAATATTCCAA |  |
|  |  |  |  |  | R: TAGGCTTCAAACCAGTCTGCTAAT |  |
| Rp_8_24014934 | Rp8-4 | 8 | 24014934 | T/C | F1: GAAGGTGACCAAGTTCATGCTGGTGAAACATAACAAAGATGGTTATATATTACT | Ambiguous |
|  |  |  |  |  | F2: GAAGGTCGGAGTCAACGGATTGGTGAAACATAACAAAGATGGTTATATATTACC |  |
|  |  |  |  |  | R: ACTGGTTCCAGTTCAATACTCCAA |  |
| Rp_8_47514582 | Rp8-5 | 8 | 47514582 | C/T | F1: GAAGGTGACCAAGTTCATGCTTAGCGAGACCAGAAAGTTCACACC | Ambiguous |
|  |  |  |  |  | F2: GAAGGTCGGAGTCAACGGATTTAGCGAGACCAGAAAGTTCACACT |  |
|  |  |  |  |  | R: CGAAAGGGGGATCAAGAGAAAGAT |  |
| Rp_8_9246580 | Rp8-6 | 8 | 9246580 | A/T | F1: GAAGGTGACCAAGTTCATGCTGCTTTCACACCTTGGCTACCA | Ambiguous |
|  |  |  |  |  | F2: GAAGGTCGGAGTCAACGGATTGCTTTCACACCTTGGCTACCT |  |
|  |  |  |  |  | R: GGAAGAAGTGGAGTATTTAGGCCA |  |
| Rp_8_28419539 | Rp8-7 | 8 | 28419539 | T/C | F1: GAAGGTGACCAAGTTCATGCTGGTTTGGATGAGATATCAACCGGT | No Polymorphism |
|  |  |  |  |  | F2: GAAGGTCGGAGTCAACGGATTGGTTTGGATGAGATATCAACCGGC |  |
|  |  |  |  |  | R: CCAAGACCAAACCGACACAAATAA |  |
| Rp_8_37712675 | Rp8-8 | 8 | 37712675 | G/C | F1: GAAGGTGACCAAGTTCATGCTGTTGGCTCCAGCTGCAAG | Success |
|  |  |  |  |  | F2: GAAGGTCGGAGTCAACGGATTGTTGGCTCCAGCTGCAAC |  |
|  |  |  |  |  | R: TATGAACGGGTTTTTATGCAGCAT |  |
| Rp_8_50778644 | Rp8-9 | 8 | 50778644 | T/C | F1: GAAGGTGACCAAGTTCATGCTGCTAGGTTGCATGGGCTAGAT | Success |
|  |  |  |  |  | F2: GAAGGTCGGAGTCAACGGATTGCTAGGTTGCATGGGCTAGAC |  |
|  |  |  |  |  | R: CCATGGAACTCGACCTAATCCATA |  |
| Rp_9_12726029 | Rp9-1 | 9 | 12726029 | G/A | F1: GAAGGTGACCAAGTTCATGCTGTAGAGAAAGGAGCGGTGATACAG | Success |
|  |  |  |  |  | F2: GAAGGTCGGAGTCAACGGATTGTAGAGAAAGGAGCGGTGATACAA |  |
|  |  |  |  |  | R: ATGTCTAAGGCTGACACTTCCATT |  |
| Rp_9_13278392 | Rp9-2 | 9 | 13278392 | A/G | F1: GAAGGTGACCAAGTTCATGCTTCTTCTTCTCTTTTTCTCTTCTTGCTA | Ambiguous |
|  |  |  |  |  | F2: GAAGGTCGGAGTCAACGGATTTCTTCTTCTCTTTTTCTCTTCTTGCTG |  |
|  |  |  |  |  | R: GACATGGAGAAAACGAAATCCGAA |  |
| Rp_9_25187830 | Rp9-3 | 9 | 25187830 | G/A | F1: GAAGGTGACCAAGTTCATGCTCCACTCAAAGAAAGACCTTCAATCAG | Ambiguous |
|  |  |  |  |  | F2: GAAGGTCGGAGTCAACGGATTCCACTCAAAGAAAGACCTTCAATCAA |  |
|  |  |  |  |  | R: TGAATTCCAAGAGGCACTGAATTG |  |
| Rp_9_42009697 | Rp9-4 | 9 | 42009697 | A/G | F1: GAAGGTGACCAAGTTCATGCTCCTCTCTGATCCCGCCAAGA | Success |
|  |  |  |  |  | F2: GAAGGTCGGAGTCAACGGATTCCTCTCTGATCCCGCCAAGG |  |
|  |  |  |  |  | R: TTACAGGGACCAAATTAACGCCTA |  |
| Rp_9_43720312 | Rp9-5 | 9 | 43720312 | T/C | F1: GAAGGTGACCAAGTTCATGCTGATGTCACAATGGCTGCGCTT | Success |
|  |  |  |  |  | F2: GAAGGTCGGAGTCAACGGATTGATGTCACAATGGCTGCGCTC |  |
|  |  |  |  |  | R: CCAGTGAGGTAGATAGCAACTGAG |  |
| Rp_10_26976618 | Rp10-1 | 10 | 26976618 | C/T | F1: GAAGGTGACCAAGTTCATGCTCGTGTAAAATATTGAGTGATAGAAAGAGC | Unsuccessful |
|  |  |  |  |  | F2: GAAGGTCGGAGTCAACGGATTCGTGTAAAATATTGAGTGATAGAAAGAGT |  |
|  |  |  |  |  | R: TCATCACCGTTTTCATCAAGCAAA |  |
| Rp_10_73634339 | Rp10-2 | 10 | 73634339 | T/G | F1: GAAGGTGACCAAGTTCATGCTGAGGGAGAATAGAAGGCAGTGTAT | Ambiguous |
|  |  |  |  |  | F2: GAAGGTCGGAGTCAACGGATTGAGGGAGAATAGAAGGCAGTGTAG |  |
|  |  |  |  |  | R: TTTTTCCGCGTGAGTTTTAGTCTG |  |
| Rp_10_849429 | Rp10-3 | 10 | 849429 | G/A | F1: GAAGGTGACCAAGTTCATGCTTGATAGTGATGATGTGACCAGTGG | No Polymorphism |
|  |  |  |  |  | F2: GAAGGTCGGAGTCAACGGATTTGATAGTGATGATGTGACCAGTGA |  |
|  |  |  |  |  | R: GGATCATTGTTCACATCTTCCACC |  |
| Rp_10_52985112 | Rp10-4 | 10 | 52985112 | T/A | F1: GAAGGTGACCAAGTTCATGCTGGGTTAAGGAGTGGAAGGATAAGT | No Polymorphism |
|  |  |  |  |  | F2: GAAGGTCGGAGTCAACGGATTGGGTTAAGGAGTGGAAGGATAAGA |  |
|  |  |  |  |  | R: CCCAATTTCAGCAATCACCATACA |  |
| Rp_10_58984753 | Rp10-5 | 10 | 58984753 | G/T | F1: GAAGGTGACCAAGTTCATGCTGGCTCCCGGTCAAGTTAAATTC | Success |
|  |  |  |  |  | F2: GAAGGTCGGAGTCAACGGATTGGCTCCCGGTCAAGTTAAATTA |  |
|  |  |  |  |  | R: TATCTGCTTCAATCCACTTGGTGA |  |
| Rp_10_41481558 | Rp10-6 | 10 | 41481558 | G/A | F1: GAAGGTGACCAAGTTCATGCTGGTGGAAATGTCCTTCGAAGAATG | Ambiguous |
|  |  |  |  |  | F2: GAAGGTCGGAGTCAACGGATTGGTGGAAATGTCCTTCGAAGAATA |  |
|  |  |  |  |  | R: AGTCACTTTAGAGGCCAACATTGA |  |
| Rp_10_44968935 | Rp10-7 | 10 | 44968935 | T/C | F1: GAAGGTGACCAAGTTCATGCTCATAGGGTCTTTCTGTTCAGGTGT | No Polymorphism |
|  |  |  |  |  | F2: GAAGGTCGGAGTCAACGGATTCATAGGGTCTTTCTGTTCAGGTGC |  |
|  |  |  |  |  | R: AGAGGCTTGGTGAAATAGACATGT |  |
| Rp_10_71873654 | Rp10-8 | 10 | 71873654 | T/C | F1: GAAGGTGACCAAGTTCATGCTGTTTGGTGTTCCCGATCTCTGA | Success |
|  |  |  |  |  | F2: GAAGGTCGGAGTCAACGGATTGTTTGGTGTTCCCGATCTCTGG |  |
|  |  |  |  |  | R: AAAGAACTCACGCATGATTTCCTG |  |
| Rp_10_73170367 | Rp10-9 | 10 | 73170367 | T/C | F1: GAAGGTGACCAAGTTCATGCTTGCTTTCTCTTCTTCTTTAGTTGATTGAT | Unsuccessful |
|  |  |  |  |  | F2: GAAGGTCGGAGTCAACGGATTTGCTTTCTCTTCTTCTTTAGTTGATTGAC |  |
|  |  |  |  |  | R: TATGAGGGTGTCCTTGTTTCGATT |  |
