# Supplementary material for: Development of KASP markers, SNP fingerprinting and population structure analysis of Robinia pseudoacacia and its closely related species
Source: Front Plant Sci. 2026 Feb 3;17:1761477. doi: 10.3389/fpls.2026.1761477 (PMC12909524; doi:10.3389/fpls.2026.1761477)
Supplement: Supplementary file 3 [file Table3.docx]

**Table S3** Genotyping results for 105 germplasms with 31 core KASP primers.

| Primer | Rp0-1 | Rp0-4 | Rp1-2 | Rp1-4 | Rp1-6 | Rp2-1 | Rp2-2 | Rp2-6 | Rp3-1 | Rp3-3 | Rp4-1 | Rp4-3 | Rp4-5 | Rp5-1 | Rp5-3 | Rp5-4 | Rp6-2 | Rp6-3 | Rp6-4 | Rp7-1 | Rp7-2 | Rp7-3 | Rp7-5 | Rp8-1 | Rp8-8 | Rp8-9 | Rp9-1 | Rp9-4 | Rp9-5 | Rp10-5 | Rp10-8 |
| --- | --- | --- | --- | --- | --- | --- | --- | --- | --- | --- | --- | --- | --- | --- | --- | --- | --- | --- | --- | --- | --- | --- | --- | --- | --- | --- | --- | --- | --- | --- | --- |
| Variation Type | A/G | T/C | G/A | T/A | T/C | G/T | A/G | A/G | T/G | C/T | T/G | G/A | G/A | T/C | A/G | G/A | C/T | G/A | C/T | A/G | C/T | A/T | C/T | T/C | G/C | T/C | G/A | A/G | T/C | G/T | T/C |
| LCX76 | A/A | T/C | A/A | T/A | T/T | T/T | A/G | ?/? | G/G | C/C | T/G | G/G | G/A | C/C | A/G | A/A | C/C | G/G | C/C | A/G | C/C | A/A | C/T | T/C | G/C | T/C | G/A | A/A | T/T | G/T | T/C |
| LCJG1 | A/A | T/C | G/A | T/A | T/T | G/T | A/A | A/G | T/T | C/T | T/T | A/A | G/G | C/C | A/G | G/G | C/C | G/G | C/C | A/G | C/T | A/A | C/C | T/C | C/C | T/C | G/G | G/G | C/C | G/G | T/T |
| ZYQS | G/G | T/C | G/G | T/T | T/T | G/G | A/G | G/G | G/G | C/C | T/G | G/G | ?/? | C/C | A/G | A/A | T/T | G/G | C/C | A/A | C/T | A/T | C/T | T/T | G/G | T/C | G/G | G/G | T/C | G/G | T/T |
| AGT | A/G | T/C | G/A | T/A | T/C | T/T | A/A | A/A | G/G | C/C | T/G | G/G | G/A | T/C | A/A | G/G | C/C | G/G | C/C | A/A | C/T | A/T | T/T | T/T | G/C | T/T | G/A | A/G | T/T | G/G | T/C |
| DCQS | G/G | T/T | G/A | T/T | T/C | G/G | A/G | A/A | T/T | C/C | T/G | A/A | G/A | T/C | A/A | G/G | T/T | G/G | C/T | A/G | C/T | A/T | C/T | T/T | G/C | T/C | G/A | A/G | T/T | G/G | T/T |
| DQS1 | G/G | C/C | G/A | T/A | T/C | G/G | A/A | A/A | T/T | T/T | T/G | G/G | G/A | T/C | A/A | G/G | C/C | G/G | C/C | A/G | C/T | T/T | T/T | T/C | G/G | C/C | G/G | A/A | T/C | G/G | T/T |
| DQS2 | A/G | T/T | G/A | T/T | T/C | G/G | A/A | A/A | T/T | C/C | T/T | A/A | G/A | T/C | A/G | G/G | C/C | G/G | C/C | A/A | C/T | A/T | C/C | T/T | G/G | T/T | G/A | A/G | T/T | G/T | T/C |
| LCSL | A/G | ?/? | G/A | T/A | T/C | G/G | A/G | G/G | T/T | C/C | T/T | G/G | A/A | T/T | A/G | G/G | C/C | G/G | C/C | A/A | C/T | T/T | C/C | C/C | G/C | T/T | G/G | A/G | T/C | G/T | C/C |
| MC87-8 | G/G | T/C | G/A | T/A | T/C | G/G | A/A | G/G | G/G | T/T | ?/? | G/G | G/G | C/C | A/A | G/G | C/C | G/G | C/C | A/G | C/T | A/A | C/T | T/T | G/G | T/T | G/A | A/G | C/C | G/T | C/C |
| MY1 | A/G | T/C | G/A | T/T | T/T | G/G | A/A | A/A | G/G | C/C | T/T | A/A | G/G | T/T | A/A | G/A | C/C | G/G | C/C | A/A | C/T | A/T | ?/? | T/T | G/G | T/C | G/A | A/G | T/C | G/G | T/T |
| CYCH | A/A | C/C | G/A | T/T | T/T | G/G | A/G | A/A | T/T | C/C | T/T | G/A | G/A | C/C | A/A | G/G | C/C | G/G | C/C | A/A | C/T | A/T | C/T | T/C | ?/? | T/C | G/A | A/G | T/C | G/G | T/C |
| LC1 | A/G | C/C | G/A | T/T | C/C | G/G | A/G | A/A | T/G | T/T | T/G | G/A | G/G | T/C | A/A | G/G | C/T | G/G | C/C | G/G | C/T | A/T | C/C | T/T | G/G | C/C | G/G | A/G | T/T | G/G | T/T |
| LC199 | A/G | C/C | G/A | T/T | T/T | G/T | A/A | G/G | T/T | T/T | T/T | G/G | G/G | T/T | A/G | G/G | C/C | A/A | T/T | A/G | C/T | A/T | T/T | T/C | G/G | T/T | G/A | A/G | T/T | G/G | T/C |
| LC10 | A/G | T/C | G/A | T/A | T/C | G/G | A/A | G/G | T/T | C/C | T/G | A/A | ?/? | T/T | A/A | G/G | T/T | G/G | C/C | G/G | C/T | A/A | C/T | T/T | G/G | C/C | A/A | A/G | T/T | G/G | T/T |
| LC100 | A/A | T/C | G/A | T/A | C/C | T/T | A/A | A/A | G/G | C/C | T/T | G/A | G/A | T/C | A/G | G/G | C/C | G/G | C/C | G/G | C/T | A/T | C/T | T/T | G/G | T/C | G/G | A/A | T/T | G/G | T/C |
| LC78-21 | A/A | C/C | G/A | T/A | ?/? | T/T | A/A | A/A | T/T | C/C | T/T | A/A | G/G | T/T | A/G | A/A | C/C | G/G | C/C | G/G | C/T | A/T | C/C | T/C | G/G | T/C | G/G | A/G | T/T | G/T | T/C |
| LCX08 | A/A | T/T | A/A | T/A | T/T | T/T | G/G | A/A | G/G | T/T | T/T | G/G | G/G | T/T | A/G | ?/? | C/T | A/A | T/T | A/G | C/T | A/T | C/T | T/C | G/G | T/C | G/A | A/A | T/C | G/T | T/C |
| MC15 | A/G | C/C | G/A | T/T | T/C | ?/? | A/A | A/A | T/T | C/T | G/G | A/A | G/A | T/T | A/A | G/G | C/C | G/G | T/T | A/G | C/T | A/T | C/T | T/C | G/G | T/C | G/G | A/A | T/C | G/G | T/T |
| ZMQS | A/G | C/C | G/A | T/T | C/C | G/G | A/A | A/A | G/G | T/T | T/T | G/G | G/A | T/T | G/G | G/G | T/T | G/G | C/C | A/G | C/T | A/T | C/C | T/C | G/G | ?/? | G/G | G/G | T/T | G/G | T/C |
| HC1 | A/G | T/T | G/A | T/T | T/T | G/G | A/A | A/A | G/G | T/T | T/T | G/A | A/A | T/C | A/A | ?/? | C/C | A/A | T/T | A/G | T/T | A/T | C/T | T/T | G/C | T/C | G/G | A/G | T/T | G/T | T/C |
| LC82-4 | A/G | C/C | G/G | T/T | C/C | G/G | A/G | A/A | T/T | T/T | T/T | G/A | G/A | T/C | A/G | G/A | C/T | G/G | C/C | G/G | C/C | A/T | C/C | T/T | G/C | T/T | G/A | G/G | T/T | G/G | T/T |
| LCX20 | A/G | T/T | G/G | T/T | T/T | G/G | A/A | G/G | T/T | C/T | T/G | G/G | G/A | T/C | A/A | A/A | C/T | G/G | C/C | A/A | T/T | A/T | C/T | T/C | G/G | T/T | G/A | A/G | T/C | T/T | T/C |
| MC16 | G/G | T/T | G/G | T/T | C/C | G/T | A/A | A/A | T/T | C/T | T/T | A/A | G/A | T/C | G/G | G/A | C/T | G/G | C/C | A/A | C/T | A/T | C/T | T/T | C/C | T/C | G/A | A/A | T/C | G/G | T/T |
| MC87-11 | A/G | T/C | G/A | T/T | T/T | G/G | A/G | A/A | T/T | C/C | T/T | G/G | G/G | T/T | A/A | G/A | C/C | G/G | C/C | G/G | C/C | A/A | C/C | C/C | G/C | T/T | G/G | A/A | T/C | G/T | T/T |
| NiuZhiCH | A/A | T/C | G/A | T/T | T/T | G/G | A/G | A/A | T/T | C/C | T/G | G/G | G/A | T/C | A/G | G/A | C/C | G/G | T/T | G/G | C/T | T/T | C/T | T/C | G/G | C/C | G/G | A/G | T/T | G/T | T/C |
| SCH | A/G | T/T | G/G | T/T | T/T | G/G | A/A | A/G | T/T | C/C | T/T | A/A | G/G | T/T | A/A | G/G | C/C | G/G | C/C | A/A | C/T | A/T | C/T | T/C | C/C | T/T | G/G | A/G | T/T | G/G | T/T |
| YCMQ1 | A/A | C/C | G/G | T/T | T/T | G/G | A/A | A/A | T/G | C/C | T/G | G/G | A/A | C/C | A/A | G/A | C/C | G/G | C/T | G/G | C/T | A/A | C/T | T/T | G/C | T/C | G/A | A/A | T/T | G/T | T/T |
| LC102 | A/A | T/C | G/A | T/A | T/C | G/G | A/A | A/A | T/G | C/T | T/T | A/A | A/A | C/C | A/A | G/A | C/C | G/G | C/C | A/A | C/C | A/A | C/T | T/T | G/C | T/T | G/G | A/A | T/T | T/T | T/T |
| LCWC | A/A | T/T | G/G | T/T | T/C | G/G | A/G | A/A | T/T | T/T | T/T | G/A | G/A | T/C | A/A | A/A | C/C | G/G | C/C | G/G | C/C | A/A | ?/? | T/T | G/G | T/T | G/G | A/A | C/C | G/T | T/T |
| MC87-1 | A/G | T/T | A/A | A/A | T/T | G/T | A/A | A/G | T/T | C/C | T/T | G/A | G/A | T/C | ?/? | G/G | C/C | G/G | C/C | G/G | C/C | A/T | C/T | T/T | G/C | T/C | G/G | A/A | T/T | G/G | T/T |
| QZ20 | A/G | C/C | G/A | T/T | T/T | G/G | A/A | A/G | T/T | C/T | T/G | G/G | G/A | T/C | A/A | G/A | C/T | G/G | C/T | A/A | C/C | A/A | C/T | T/T | G/G | T/C | A/A | A/G | T/T | T/T | T/T |
| J10 | A/A | T/C | G/A | T/T | T/C | G/G | A/G | G/G | T/T | C/C | T/G | G/G | G/A | T/C | A/A | A/A | C/C | A/A | C/C | G/G | C/T | A/A | T/T | T/T | G/G | T/T | G/A | A/A | T/C | G/G | T/T |
| LC104 | A/A | C/C | G/G | T/T | T/T | T/T | A/A | G/G | T/T | C/T | T/T | G/A | G/G | T/T | A/A | A/A | C/C | A/A | C/C | A/A | C/T | A/T | C/C | T/T | G/G | T/C | G/A | A/G | T/T | G/G | T/T |
| LC13 | A/A | T/T | G/G | T/A | C/C | G/G | A/A | G/G | T/G | C/C | T/T | G/G | G/G | T/T | A/A | G/A | C/C | G/G | C/C | G/G | C/T | T/T | T/T | T/C | G/G | T/C | G/A | A/A | T/C | G/G | T/T |
| LC221 | A/G | T/T | G/G | T/T | T/C | G/G | A/G | G/G | T/T | C/T | ?/? | G/A | A/A | C/C | A/G | G/G | C/T | G/A | C/C | G/G | C/C | A/T | C/T | T/C | C/C | T/T | G/A | A/A | T/T | G/G | T/T |
| LC42 | G/G | T/C | G/A | T/T | C/C | G/G | A/A | A/A | T/T | C/C | T/G | G/A | G/A | T/C | A/G | G/A | T/T | G/G | T/T | G/G | C/C | A/A | C/T | T/T | ?/? | T/C | A/A | A/G | T/T | G/T | T/C |
| LC68 | A/G | C/C | G/A | T/T | C/C | T/T | A/G | A/A | G/G | C/T | T/G | G/G | G/G | T/T | A/A | G/A | C/C | G/G | C/C | G/G | C/C | A/T | ?/? | T/T | G/G | T/C | A/A | A/G | T/T | G/G | T/T |
| LC8217 | A/A | T/C | A/A | T/A | T/T | T/T | A/G | A/A | T/G | C/T | T/G | G/G | G/A | T/C | A/G | A/A | C/T | G/G | C/C | G/G | C/T | A/A | C/T | T/C | G/C | T/C | G/A | A/A | T/T | G/T | T/C |
| LYZBQS | A/A | T/C | G/A | T/T | T/T | G/G | A/G | A/A | T/T | C/C | T/G | G/G | G/A | T/C | A/G | G/A | C/C | G/G | T/T | G/G | C/C | T/T | C/T | T/C | G/G | C/C | G/G | A/G | T/T | G/T | T/C |
| LC12 | A/G | C/C | G/G | T/T | C/C | G/G | A/G | A/A | T/T | C/T | T/G | G/G | G/A | T/C | A/G | G/A | C/T | G/G | C/C | G/G | C/C | A/A | C/T | T/C | G/C | T/T | G/G | G/G | T/C | G/G | T/T |
| LC15 | A/G | T/T | G/A | T/A | T/T | G/G | A/A | A/A | T/T | C/C | T/T | G/A | G/G | T/T | A/A | G/G | C/C | G/G | T/T | A/A | C/T | A/T | C/C | T/C | G/C | T/C | G/A | A/A | T/C | G/G | T/T |
| LC23 | A/A | T/C | G/G | T/T | T/T | G/G | A/A | A/A | G/G | C/T | T/T | G/A | G/A | T/C | A/G | G/A | C/C | G/G | C/C | A/A | C/T | A/T | C/T | T/C | G/G | T/C | G/G | A/G | T/T | G/G | T/T |
| LC235 | A/G | T/C | G/G | T/T | T/T | G/G | A/G | A/A | T/G | T/T | T/G | A/A | G/G | T/T | A/A | G/G | C/T | G/G | T/T | A/A | C/T | A/T | C/T | T/T | G/G | T/T | G/G | A/G | T/T | G/G | T/C |
| LYH | A/A | T/C | G/A | T/T | T/T | G/G | A/G | A/A | T/T | C/C | T/G | G/G | G/A | T/C | A/G | G/A | C/T | G/G | T/T | G/G | C/C | ?/? | C/T | T/C | G/G | C/C | G/G | A/G | T/T | G/T | T/C |
| MZA | A/G | T/T | A/A | T/T | T/C | G/G | A/A | A/A | T/T | C/T | T/T | G/G | G/A | T/C | ?/? | A/A | C/C | G/G | C/T | G/G | C/T | T/T | C/T | T/T | G/C | T/T | G/G | A/A | T/T | G/G | T/T |
| LC11 | A/G | T/T | G/A | T/T | T/T | G/G | A/A | A/A | T/T | C/C | T/T | G/G | G/A | T/C | A/G | G/A | C/C | G/G | C/C | A/A | C/T | A/T | C/T | T/C | C/C | C/C | G/G | A/A | T/T | G/G | T/T |
| LCHH | A/G | T/C | G/A | T/T | C/C | G/G | A/A | A/A | T/T | C/T | T/T | G/A | A/A | C/C | A/A | G/G | C/C | G/G | C/C | G/G | C/C | A/T | C/T | T/T | C/C | T/C | G/A | A/G | T/C | G/T | T/C |
| LCZY | A/A | T/C | A/A | T/T | T/C | G/G | A/G | A/A | G/G | C/C | T/T | G/G | ?/? | ?/? | A/A | G/A | T/T | A/A | T/T | A/A | C/C | T/T | C/T | T/C | G/C | T/T | ?/? | A/A | T/C | G/G | T/T |
| QZ6 | A/A | T/C | A/A | T/A | C/C | G/T | A/A | A/A | T/G | C/C | T/T | G/A | G/A | T/C | A/G | G/A | C/T | ?/? | C/T | G/G | C/C | A/T | C/T | T/C | ?/? | T/T | G/A | G/G | T/T | G/T | C/C |
| JYCH | G/G | C/C | G/G | T/A | T/C | G/T | A/G | G/G | T/T | ?/? | T/T | G/A | G/G | T/T | A/G | G/A | ?/? | G/G | C/C | A/A | C/C | A/T | C/C | T/T | G/G | T/C | G/G | A/A | T/C | G/G | T/C |
| LC103 | A/A | T/T | G/A | T/T | T/C | G/G | A/A | A/A | T/G | C/T | T/T | G/G | G/A | T/C | A/A | A/A | C/T | A/A | T/T | A/A | C/T | A/T | C/C | T/C | C/C | T/T | G/A | A/A | T/C | G/G | C/C |
| LC32 | A/G | T/T | G/G | T/T | T/T | G/G | A/A | G/G | T/T | T/T | T/T | G/G | G/A | T/C | A/A | G/A | C/C | A/A | C/C | A/A | C/T | A/T | C/T | T/T | G/G | T/T | G/A | A/G | C/C | G/G | T/T |
| LCJSY1 | A/A | T/T | G/A | T/A | C/C | G/G | A/A | G/G | T/G | C/T | T/T | G/G | G/A | T/C | A/A | G/A | ?/? | G/G | C/C | G/G | C/T | A/T | C/C | T/T | G/G | T/T | G/A | G/G | T/T | G/T | T/C |
| LCX32 | A/A | T/C | A/A | T/A | T/T | G/G | A/G | A/A | T/T | C/C | T/T | G/G | G/A | T/C | A/G | G/A | C/C | A/A | T/T | A/A | C/C | A/A | C/C | T/C | G/G | T/T | G/G | G/G | T/T | G/T | T/T |
| QSNZ | A/G | C/C | G/A | T/A | T/T | T/T | A/G | G/G | T/T | C/T | T/G | G/G | G/A | T/C | A/G | A/A | C/T | G/G | C/C | G/G | C/C | A/T | C/T | T/T | G/C | T/C | G/A | A/G | T/T | G/T | T/T |
| YC8005 | A/G | T/C | G/G | T/T | T/T | G/G | G/G | A/A | T/T | C/T | T/T | G/G | A/A | C/C | A/A | G/G | C/C | A/A | T/T | A/A | C/C | A/T | C/C | C/C | G/G | T/T | G/A | G/G | T/T | G/G | T/T |
| J3 | A/G | T/C | G/G | T/A | C/C | G/G | A/G | A/G | T/G | C/C | T/T | G/G | G/G | T/T | A/G | G/G | C/C | G/G | C/C | A/A | C/C | T/T | C/C | T/C | C/C | T/C | G/G | A/A | T/C | G/T | T/T |
| QZ8 | A/G | T/T | G/G | T/T | T/T | G/G | A/A | G/G | T/T | T/T | T/T | G/G | G/A | T/C | A/A | G/A | C/T | G/G | C/C | A/A | C/C | A/A | C/T | T/T | G/C | T/T | ?/? | A/A | C/C | G/G | T/T |
| BL-5 | A/A | T/C | G/G | T/A | T/C | T/T | A/G | G/G | T/G | C/T | T/T | G/A | G/A | T/C | A/A | G/G | C/C | G/G | C/C | A/A | C/C | A/T | C/T | T/C | G/G | T/C | G/G | A/G | T/T | G/G | T/T |
| LC166 | A/G | C/C | A/A | T/T | T/T | T/T | A/G | G/G | G/G | T/T | T/T | G/A | G/A | T/C | A/G | G/A | T/T | G/G | C/C | G/G | C/C | A/A | C/C | T/C | G/C | ?/? | G/A | ?/? | T/T | G/T | T/T |
| LJ29 | A/A | T/C | A/A | T/T | T/T | G/G | G/G | A/A | T/T | C/C | T/T | G/A | G/A | T/C | A/G | G/G | C/T | G/G | C/C | A/A | C/C | A/T | C/T | T/C | C/C | T/T | G/G | A/A | T/C | G/G | T/C |
| QZ12 | G/G | C/C | G/G | T/A | T/C | G/G | A/G | A/A | T/T | C/T | T/T | A/A | G/A | T/C | A/A | G/G | C/C | G/G | C/C | G/G | C/C | A/A | T/T | T/T | G/G | C/C | G/A | G/G | T/T | G/T | T/T |
| QZ2 | A/G | C/C | G/A | T/T | T/C | G/G | A/A | A/G | T/T | C/T | T/G | A/A | A/A | C/C | A/A | G/G | T/T | G/G | C/C | G/G | C/C | A/T | C/C | T/C | G/C | C/C | G/A | G/G | T/C | G/T | T/T |
| LC20 | A/A | T/T | G/G | T/T | T/T | G/G | A/A | A/A | T/T | C/C | T/T | G/A | G/A | T/C | A/G | ?/? | C/C | G/G | C/C | A/A | C/C | T/T | C/C | T/T | G/G | T/C | G/A | A/G | T/C | T/T | T/T |
| LC820026 | A/G | C/C | G/G | T/A | T/T | G/G | A/A | A/A | T/T | C/T | T/G | G/A | G/G | T/T | A/A | G/A | C/T | A/A | T/T | G/G | C/T | A/A | C/C | T/T | ?/? | T/C | G/A | A/G | T/T | T/T | T/T |
| LC90 | G/G | T/T | G/G | T/A | T/C | G/G | A/A | G/G | T/T | C/T | T/G | G/G | G/A | T/C | A/A | G/A | T/T | G/G | C/C | A/A | C/C | A/T | C/T | T/T | G/C | T/T | G/G | A/G | C/C | G/T | T/T |
| LCJG2 | A/A | T/C | G/A | T/T | T/T | G/G | A/G | G/G | T/G | C/T | T/G | G/A | G/G | T/T | A/A | G/G | C/C | G/G | C/C | G/G | C/C | A/T | C/T | T/T | G/C | T/C | G/A | A/G | T/T | G/G | T/C |
| SCWG | G/G | C/C | A/A | T/T | T/T | G/G | A/A | G/G | T/G | C/T | T/T | G/A | A/A | C/C | A/A | G/G | C/T | G/G | C/C | A/A | C/T | ?/? | T/T | T/C | G/C | T/C | G/G | A/A | T/T | G/T | T/T |
| LC27 | A/A | T/C | G/A | T/A | C/C | T/T | A/G | A/A | T/G | C/T | T/T | G/G | A/A | C/C | A/G | G/A | C/C | A/A | T/T | G/G | C/T | A/T | T/T | T/T | ?/? | T/C | G/G | A/G | T/C | T/T | C/C |
| LC51 | G/G | C/C | G/G | T/T | T/C | G/G | A/A | A/A | T/T | C/T | T/T | G/G | A/A | C/C | A/A | A/A | C/C | G/G | C/C | G/G | C/C | A/T | C/C | T/C | G/C | T/C | G/G | A/A | T/C | G/T | T/T |
| LCJN1 | A/A | C/C | G/G | T/A | T/T | G/G | A/A | A/A | T/G | C/C | T/G | G/G | A/A | C/C | A/A | G/A | C/C | G/G | C/T | G/G | C/C | A/A | C/T | T/T | G/C | T/C | G/A | A/A | T/T | G/T | T/T |
| QZ9 | A/A | T/T | G/A | T/A | T/T | T/T | A/G | A/A | T/G | C/T | T/T | G/A | G/A | T/C | A/A | G/A | C/C | G/G | C/C | A/A | C/C | A/A | C/T | T/T | G/C | T/T | G/G | G/G | T/C | G/T | T/T |
| WCWG | A/A | T/C | G/G | T/A | T/C | T/T | A/G | A/G | T/G | C/T | T/T | G/A | G/A | T/C | A/A | G/G | C/C | G/G | C/C | A/A | C/C | A/T | ?/? | T/C | G/G | T/C | G/G | A/G | T/T | G/G | T/T |
| LC200 | A/A | C/C | G/A | T/A | T/T | G/T | G/G | A/A | T/T | C/C | T/T | G/G | G/G | T/T | A/G | G/A | C/C | A/A | C/C | G/G | C/C | A/T | C/T | T/T | G/G | C/C | G/A | A/A | T/T | G/G | T/C |
| LC50 | A/G | T/C | G/A | T/A | T/C | G/G | A/A | G/G | T/T | T/T | T/T | G/A | G/A | T/C | A/A | A/A | C/T | G/G | C/C | A/A | C/C | A/T | C/C | T/T | G/C | T/C | A/A | A/G | T/C | T/T | T/T |
| LC7 | G/G | T/C | G/A | T/T | T/C | T/T | A/A | G/G | T/G | C/C | T/T | G/G | G/G | T/T | A/A | G/A | C/T | G/G | C/C | G/G | C/C | A/A | C/C | T/T | C/C | C/C | G/A | G/G | T/T | G/T | T/T |
| LC820027 | A/A | T/C | G/G | A/A | T/C | ?/? | A/G | G/G | T/T | C/C | T/T | G/G | A/A | C/C | ?/? | A/A | C/C | G/G | C/C | G/G | C/C | A/T | C/C | T/C | C/C | T/C | G/A | A/A | C/C | G/T | T/T |
| LCX04 | G/G | C/C | G/G | T/A | T/T | G/G | A/G | G/G | T/T | C/C | T/T | G/G | G/G | T/T | A/A | A/A | C/T | G/G | C/C | G/G | C/C | A/T | C/T | T/T | G/C | T/C | G/G | A/G | T/T | G/T | T/C |
| LMQS | G/G | T/T | G/G | T/A | T/C | G/G | A/A | A/A | T/T | C/T | T/G | G/G | G/A | T/C | A/G | G/A | C/T | G/G | C/C | G/G | C/C | T/T | T/T | T/T | G/G | T/T | A/A | A/G | T/C | G/T | ?/? |
| LC93 | A/G | T/T | A/A | T/A | T/C | G/G | A/A | A/A | G/G | C/T | T/T | G/G | A/A | C/C | A/A | G/A | C/C | G/G | T/T | G/G | C/C | A/T | C/T | T/T | G/G | T/T | G/A | A/A | T/C | G/T | C/C |
| JCAB | A/G | T/C | A/A | T/A | T/T | T/T | A/G | A/A | G/G | C/C | T/T | G/G | G/A | T/C | A/G | A/A | C/C | A/A | T/T | G/G | C/C | A/A | C/T | T/C | G/C | T/T | G/G | A/G | T/C | G/T | T/T |
| LC64 | G/G | T/T | G/G | T/A | T/C | G/G | A/A | G/G | T/T | C/T | T/T | G/G | G/A | T/C | A/A | G/A | T/T | G/G | C/C | A/A | C/C | A/T | C/T | T/T | G/C | T/T | G/G | ?/? | C/C | G/T | T/T |
| LC85 | A/G | C/C | G/A | T/T | T/C | G/G | A/G | G/G | T/G | C/C | T/G | G/G | A/A | C/C | A/A | G/A | C/C | G/G | T/T | G/G | C/C | A/T | C/T | T/T | G/C | T/C | A/A | A/G | T/T | G/T | C/C |
| LC59 | A/A | T/C | G/G | T/A | T/C | T/T | A/G | A/A | G/G | C/T | T/G | G/G | G/G | T/T | G/G | G/A | C/T | G/G | C/T | G/G | C/C | A/T | C/C | T/C | G/G | T/C | G/G | A/G | T/C | G/G | T/T |
| LC79 | A/G | ?/? | G/A | T/A | C/C | T/T | A/A | G/G | T/G | C/T | T/G | ?/? | G/A | T/C | A/G | G/G | C/C | G/G | C/C | G/G | C/C | A/T | C/C | T/T | G/C | T/T | G/A | A/G | T/C | G/G | T/T |
| LCX37 | A/G | T/C | G/G | T/A | C/C | G/G | A/G | A/A | T/T | C/T | T/T | G/A | G/G | T/T | A/G | G/A | C/C | G/G | C/C | A/A | C/C | A/A | C/C | T/T | G/G | T/T | G/G | A/A | T/C | G/G | C/C |
| QZ27 | A/G | T/T | G/A | T/A | T/T | G/T | A/A | G/G | G/G | C/C | T/G | G/G | G/G | T/T | A/A | G/A | C/T | G/G | C/C | A/A | C/C | A/A | C/T | T/T | G/C | T/C | A/A | G/G | T/T | G/G | T/C |
| MC87-10 | A/A | T/C | G/G | T/T | T/C | G/G | A/A | G/G | T/T | C/T | T/T | G/A | G/G | T/T | A/A | A/A | C/C | G/G | C/C | A/G | C/C | T/T | C/T | T/T | G/G | T/C | G/G | A/G | T/T | G/T | ?/? |
| NianZhiCH | A/A | C/C | G/G | T/T | ?/? | G/G | A/G | A/A | T/T | C/C | T/G | G/G | G/A | T/C | G/G | A/A | C/T | G/G | C/T | A/A | C/C | A/T | T/T | T/T | C/C | T/T | G/G | A/G | T/C | G/G | T/T |
| JCS10 | A/A | T/C | G/A | T/T | T/C | G/G | A/G | G/G | T/T | C/C | T/G | ?/? | G/A | T/C | A/A | A/A | C/C | A/A | C/C | G/G | C/C | A/A | T/T | T/T | G/G | T/T | G/A | A/A | T/C | G/G | T/T |
| LC155 | G/G | T/T | G/G | T/A | T/C | G/G | A/A | A/A | T/T | C/T | T/G | G/G | G/A | T/C | A/G | G/A | C/T | G/G | C/C | G/G | C/T | T/T | T/T | T/T | G/G | T/T | A/A | A/G | T/C | G/T | T/C |
| DHZY | ?/? | C/C | G/G | T/T | T/T | T/T | A/A | A/A | G/G | C/C | T/T | G/G | G/G | T/T | A/G | G/A | C/T | G/A | T/T | G/G | C/C | A/T | C/T | T/T | G/G | C/C | G/G | A/G | C/C | T/T | ?/? |
| LC2 | A/G | T/T | A/A | A/A | T/C | G/T | A/G | A/A | T/T | C/C | T/T | G/G | G/G | T/T | A/A | A/A | T/T | G/G | C/T | G/G | C/C | A/A | T/T | T/T | G/C | T/T | A/A | A/G | T/T | G/T | C/C |
| LC26 | A/A | C/C | G/G | T/T | T/C | G/G | A/A | A/A | T/T | C/C | T/T | A/A | G/A | T/C | A/A | G/G | C/T | G/G | C/C | G/G | C/C | ?/? | C/T | T/C | G/C | T/C | G/A | A/G | T/C | G/G | T/C |
| LC40 | A/G | ?/? | G/A | T/T | T/C | G/G | A/A | G/G | T/T | T/T | T/T | G/G | G/G | T/T | A/A | G/A | C/T | G/G | C/C | G/G | C/C | A/T | C/T | T/T | G/C | T/C | G/A | G/G | T/C | G/G | T/T |
| LCX44 | G/G | C/C | G/G | T/A | T/C | G/G | A/G | A/A | T/G | T/T | T/G | G/G | G/A | T/C | A/G | G/G | C/T | G/G | C/C | G/G | C/C | A/T | C/T | T/T | G/G | T/C | G/A | A/G | T/C | G/G | T/C |
| XMXG | A/G | T/C | A/A | T/A | T/T | T/T | A/G | A/A | G/G | C/C | T/T | G/G | G/A | T/C | A/G | A/A | C/C | A/A | T/T | G/G | C/C | A/A | C/T | T/C | G/C | T/T | G/G | G/G | T/C | G/T | T/T |
| DYHH | A/A | C/C | G/G | T/T | T/T | T/T | A/A | A/A | G/G | C/C | T/T | G/G | G/G | T/T | A/G | G/A | C/T | G/A | T/T | G/G | C/C | A/T | C/T | T/T | G/C | C/C | G/G | A/G | C/C | T/T | T/T |
| LC117 | A/G | T/T | G/G | T/A | T/T | T/T | A/A | G/G | T/G | C/C | T/T | A/A | G/A | T/C | A/G | A/A | C/T | G/G | C/C | A/A | C/C | A/T | C/T | T/T | G/G | T/C | G/G | A/G | T/T | G/G | T/T |
| XHH | A/A | C/C | G/G | T/A | ?/? | T/T | A/A | A/A | G/G | C/C | T/T | G/G | G/G | T/T | A/G | ?/? | C/T | G/A | T/T | G/G | C/C | A/T | C/T | T/C | G/G | C/C | G/G | A/G | C/C | T/T | T/T |
| DYCH | A/G | T/T | G/G | T/T | T/C | T/T | A/A | A/A | T/T | T/T | T/G | G/A | G/G | T/T | A/G | G/G | C/C | G/G | C/C | A/A | C/C | A/T | C/T | T/C | G/G | T/T | G/A | A/G | T/C | T/T | T/T |
| LC179 | A/A | T/C | G/G | T/A | T/T | G/G | A/A | G/G | T/G | C/T | T/T | G/A | ?/? | ?/? | A/A | G/G | ?/? | G/G | C/C | G/G | T/T | A/T | C/T | T/C | G/G | C/C | G/G | A/A | T/T | G/G | T/T |
| LC63 | G/G | T/T | G/G | T/T | C/C | G/T | A/A | A/A | T/T | C/T | T/T | A/A | G/A | T/C | G/G | G/A | C/T | G/G | C/C | A/A | C/C | A/T | C/T | T/T | C/C | T/C | G/A | A/A | T/C | G/G | T/T |
| XMXGJX | A/A | T/C | G/G | T/A | ?/? | G/G | G/G | G/G | T/G | C/C | G/G | G/G | G/G | T/T | A/A | A/A | C/C | G/G | C/C | A/A | C/T | T/T | C/C | T/C | G/G | ?/? | G/G | G/G | T/C | G/G | T/T |
| FW | A/A | C/C | G/G | T/T | ?/? | G/G | G/G | A/A | T/G | C/T | G/G | G/G | G/G | T/T | ?/? | A/A | T/T | G/G | T/T | A/A | C/C | T/T | C/C | T/T | G/C | ?/? | G/G | G/G | C/C | G/G | ?/? |
