# Supplementary material for: Development of KASP markers, SNP fingerprinting and population structure analysis of Robinia pseudoacacia and its closely related species
Source: Front Plant Sci. 2026 Feb 3;17:1761477. doi: 10.3389/fpls.2026.1761477 (PMC12909524; doi:10.3389/fpls.2026.1761477)
Supplement: Supplementary file 5 [file Table5.docx]

**Table S5** Validation results of 31 core KASP primer.

| Prime | Rp0-1 | Rp0-4 | Rp1-2 | Rp1-4 | Rp1-6 | Rp2-1 | Rp2-2 | Rp2-6 | Rp3-1 | Rp3-3 | Rp4-1 | Rp4-3 | Rp4-5 | Rp5-1 | Rp5-3 | Rp5-4 | Rp6-2 | Rp6-3 | Rp6-4 | Rp7-1 | Rp7-2 | Rp7-3 | Rp7-5 | Rp8-1 | Rp8-8 | Rp8-9 | Rp9-1 | Rp9-4 | Rp9-5 | Rp10-5 | Rp10-8 |
| --- | --- | --- | --- | --- | --- | --- | --- | --- | --- | --- | --- | --- | --- | --- | --- | --- | --- | --- | --- | --- | --- | --- | --- | --- | --- | --- | --- | --- | --- | --- | --- |
| Variation Type | A/G | T/C | G/A | T/A | T/C | G/T | A/G | A/G | T/G | C/T | T/G | G/A | G/A | T/C | A/G | G/A | C/T | G/A | C/T | A/G | C/T | A/T | C/T | T/C | G/C | T/C | G/A | A/G | T/C | G/T | T/C |
| 133×MQ2 | A/G | T/T | G/G | T/A | T/C | ?/? | A/G | A/A | T/G | C/T | T/T | G/A | G/G | T/C | A/A | G/G | C/C | G/G | C/C | G/G | C/T | A/A | C/T | T/T | G/G | T/T | G/G | A/G | T/C | T/T | T/T |
| 152×6815-2 | A/G | T/T | G/G | T/T | C/C | G/T | A/A | ?/? | T/T | C/C | T/G | G/A | G/A | C/C | A/G | G/G | C/C | G/G | C/C | A/A | C/T | A/A | C/T | T/T | G/G | T/T | G/G | A/A | T/C | G/T | T/C |
| 133×MQ215-5 | A/A | T/C | G/G | T/T | T/T | G/T | A/G | G/G | T/G | C/C | T/T | G/G | G/G | T/C | A/A | A/A | C/C | A/A | C/C | ?/? | C/T | A/A | T/T | T/C | G/C | T/T | G/A | A/G | T/C | T/T | T/T |
| 15-63 | A/G | T/C | G/G | T/T | T/C | T/T | A/A | G/G | T/G | C/C | T/T | A/A | G/G | T/T | A/A | A/A | C/C | A/A | T/T | A/A | C/C | A/T | C/C | T/T | C/C | C/C | G/G | G/G | T/T | G/G | T/T |
| 07×X2515-1 | A/G | T/C | G/G | T/T | T/T | T/T | A/G | G/G | T/T | C/C | T/G | G/G | G/G | T/T | A/G | G/G | C/C | A/A | C/C | G/G | C/C | A/A | C/C | T/T | G/G | C/C | G/A | A/G | T/T | G/G | T/T |
| X1×4215-1 | G/G | C/C | G/G | T/T | T/C | G/G | A/A | A/A | T/G | C/C | T/T | G/G | ?/? | T/T | A/G | G/G | C/T | G/G | C/T | G/G | C/T | A/A | C/C | T/T | G/G | T/T | G/A | A/A | T/T | G/G | T/C |
| 133×DH15-4 | A/G | T/C | A/A | T/T | T/T | G/G | A/G | A/A | T/G | C/C | T/T | G/G | G/G | C/C | A/G | G/G | C/C | G/G | T/T | G/G | C/C | A/A | C/T | T/T | G/G | T/T | G/G | A/G | C/C | G/T | T/T |
| 229×J415-10 | A/G | T/C | G/G | T/A | T/C | G/T | A/G | G/G | T/T | C/T | T/T | G/A | G/G | T/C | A/A | G/G | C/C | G/G | T/T | A/A | C/C | T/T | C/T | T/T | ?/? | T/C | A/A | A/A | C/C | ?/? | T/T |
| HC15-25 | A/G | T/C | G/G | T/T | T/C | G/G | A/G | A/A | T/T | C/T | ?/? | G/G | G/G | T/T | A/A | G/G | C/C | G/G | C/C | ?/? | C/T | ?/? | C/T | T/C | G/G | T/T | G/G | A/G | C/C | G/G | T/T |
| 133×DH15-5 | A/G | T/T | A/A | T/A | T/T | G/G | A/A | A/A | T/T | C/C | T/T | G/G | G/G | T/T | A/A | G/G | C/C | G/G | C/C | A/A | C/T | A/T | C/T | T/C | G/G | T/C | G/A | A/G | T/C | G/T | T/T |
| 57×X1515-24 | A/G | C/C | G/G | A/A | T/C | G/T | A/G | G/G | T/T | C/T | T/T | G/G | A/A | T/T | A/A | G/G | T/T | G/G | C/C | A/A | C/T | A/T | T/T | T/T | G/G | C/C | G/A | A/G | T/T | G/G | T/T |
| 57×X1515-39 | A/G | T/C | G/G | A/A | T/T | T/T | A/G | G/G | T/T | C/T | T/T | G/A | A/A | ?/? | G/G | G/G | T/T | G/G | C/C | ?/? | C/C | A/A | C/T | T/T | G/G | C/C | A/A | A/A | T/T | T/T | T/T |
| 57×X1515-9 | A/G | C/C | G/G | T/A | T/C | G/T | A/A | G/G | T/T | C/T | T/T | G/A | G/A | C/C | G/G | G/G | T/T | G/G | C/C | A/A | C/T | T/T | T/T | T/T | G/G | T/C | G/A | A/G | T/T | T/T | T/T |
| 29J415-21 | G/G | T/T | G/G | T/T | T/C | G/T | A/G | A/A | T/T | C/T | T/T | G/G | G/G | T/C | A/G | G/G | C/C | G/G | T/T | A/A | C/T | A/A | C/T | T/T | G/G | T/C | G/A | A/A | C/C | G/G | T/T |
| 152×6215-17 | A/G | T/T | G/G | T/T | T/T | G/G | A/A | A/A | T/G | ?/? | T/T | A/A | G/A | T/T | A/G | A/A | C/C | G/G | C/C | A/A | T/T | A/T | C/T | T/T | G/G | T/T | G/G | A/A | T/C | G/G | T/C |
| X46GC15-1 | G/G | T/T | G/G | T/T | T/T | G/G | A/G | A/A | T/T | C/C | T/G | G/G | G/A | C/C | G/G | A/A | C/C | G/G | T/T | G/G | C/T | A/T | T/T | T/T | G/G | C/C | G/G | A/A | T/T | G/G | T/C |
| DH4215-01 | A/G | T/C | G/G | T/T | C/C | G/G | A/A | A/A | T/T | C/C | T/G | G/A | G/G | T/T | G/G | A/A | T/T | G/G | T/T | G/G | C/C | A/T | T/T | T/T | G/G | ?/? | A/A | A/A | T/C | ?/? | T/C |
| 152×J415-1 | A/G | T/C | G/G | T/A | T/C | T/T | A/G | A/A | T/T | C/T | T/T | G/G | G/A | T/T | A/G | G/G | C/C | G/G | T/T | ?/? | C/C | ?/? | C/T | T/T | G/G | T/C | A/A | A/A | C/C | G/G | T/T |
| 152×1015-4 | A/G | T/T | G/G | T/T | T/C | G/G | A/A | G/G | T/T | C/C | T/G | A/A | A/A | T/T | A/G | G/G | C/C | G/G | C/C | A/G | C/C | A/A | T/T | T/T | G/G | T/C | G/A | A/A | T/T | G/G | T/T |
| 152×1015-21 | A/G | C/C | G/G | T/T | T/C | T/T | A/A | A/A | T/T | C/C | T/T | G/G | G/A | C/C | ?/? | G/G | C/C | G/G | C/C | ?/? | C/T | A/A | C/T | T/T | G/G | T/T | G/G | A/A | T/C | G/G | T/T |
| 152×J415-3 | A/G | C/C | G/G | T/T | C/C | T/T | A/G | A/A | T/T | C/T | T/T | G/G | A/A | T/T | A/G | G/G | C/C | G/G | C/C | A/A | C/C | A/T | C/T | T/T | G/G | T/T | G/G | A/A | C/C | G/G | T/T |
| 80-102×02ZC15-1 | A/A | T/C | G/G | T/A | T/C | G/G | A/A | G/G | T/G | C/T | T/T | G/A | G/G | T/T | ?/? | G/G | C/C | G/G | T/T | G/G | C/T | T/T | T/T | T/T | G/G | T/T | G/A | A/G | T/T | G/G | T/C |
| 13ZC05-1 | A/G | T/T | G/G | T/T | T/C | G/G | A/G | A/A | T/T | C/C | T/G | G/G | G/G | T/T | A/A | G/G | T/T | G/G | C/C | G/G | C/T | A/A | T/T | T/T | G/G | T/T | G/A | A/G | T/C | G/G | T/T |
| 07ZC05-03 | A/G | T/C | G/G | T/T | T/T | T/T | A/A | G/G | T/T | C/C | T/T | G/G | G/G | T/T | A/A | G/G | T/T | G/G | C/T | G/G | C/T | A/A | C/T | T/T | C/C | T/C | A/A | G/G | T/C | T/T | T/T |
| HC15-3 | A/A | C/C | G/G | T/T | T/T | T/T | A/G | A/G | T/T | ?/? | T/T | G/G | A/A | C/C | A/G | G/G | C/C | A/A | C/T | A/A | C/T | A/T | C/C | T/C | G/G | T/C | G/G | A/G | T/T | G/G | T/T |
| X9 | A/G | T/C | G/G | T/T | T/T | G/T | A/G | A/A | T/G | C/T | T/G | G/A | A/A | T/T | A/A | G/G | T/T | G/G | C/C | G/G | C/T | T/T | C/C | T/T | G/G | T/T | G/G | A/A | C/C | G/G | T/C |
| 42×DH15-1 | A/G | T/C | G/G | T/T | T/T | G/G | A/G | G/G | T/T | C/C | T/T | G/G | A/A | T/T | A/A | A/A | C/C | G/G | C/C | A/A | C/C | ?/? | C/T | T/T | G/C | T/T | A/A | A/A | T/C | G/G | T/T |
| 42×DH15-2 | A/G | C/C | G/G | T/T | ?/? | G/G | A/A | A/A | T/G | C/C | T/T | G/G | G/A | T/C | G/G | G/G | T/T | A/A | ?/? | G/G | C/C | ?/? | C/C | T/T | G/G | C/C | G/A | G/G | T/C | G/G | T/C |
| 80-10×01ZC1501 | A/A | T/C | G/G | T/T | C/C | G/G | A/A | ?/? | T/T | C/C | T/G | G/A | A/A | T/C | A/A | G/G | C/C | G/G | C/C | G/G | T/T | ?/? | C/T | T/T | G/C | T/C | G/A | A/G | T/C | G/G | T/T |
| X5 | A/A | T/C | A/A | T/A | T/C | G/G | A/G | A/A | T/T | C/C | T/T | G/G | A/A | T/C | A/G | G/G | C/C | G/G | C/C | A/A | C/T | T/T | C/T | T/C | G/G | T/C | G/G | G/G | T/C | G/G | T/T |
